# Gene regulatory networks modelling using a dynamic evolutionary hybrid

**DOI:** 10.1186/1471-2105-11-140

**Published:** 2010-03-18

**Authors:** Ioannis A Maraziotis, Andrei Dragomir, Dimitris Thanos

**Affiliations:** 1 Institute of Molecular Biology, Genetics and Biotechnology, Biomedical Research Foundation, Academy of Athens, 4 Soranou Efesiou Street, Athens 11527, Greece; 2 Harrington Department of Bioengineering, Arizona State University, Tempe, AZ, USA

## Abstract

**Background:**

Inference of gene regulatory networks is a key goal in the quest for understanding fundamental cellular processes and revealing underlying relations among genes. With the availability of gene expression data, computational methods aiming at regulatory networks reconstruction are facing challenges posed by the data's high dimensionality, temporal dynamics or measurement noise. We propose an approach based on a novel multi-layer evolutionary trained neuro-fuzzy recurrent network (ENFRN) that is able to select potential regulators of target genes and describe their regulation type.

**Results:**

The recurrent, self-organizing structure and evolutionary training of our network yield an optimized pool of regulatory relations, while its fuzzy nature avoids noise-related problems. Furthermore, we are able to assign scores for each regulation, highlighting the confidence in the retrieved relations. The approach was tested by applying it to several benchmark datasets of yeast, managing to acquire biologically validated relations among genes.

**Conclusions:**

The results demonstrate the effectiveness of the ENFRN in retrieving biologically valid regulatory relations and providing meaningful insights for better understanding the dynamics of gene regulatory networks.

The algorithms and methods described in this paper have been implemented in a Matlab toolbox and are available from: http://bioserver-1.bioacademy.gr/DataRepository/Project_ENFRN_GRN/.

## Background

Fundamental biological processes including cell differentiation, metabolism, the cell cycle or signal transduction are governed by the expression of genes. Gene products and especially the amounts and temporal patterns in which these products are expressed in the cell enable cell survival and numerous cellular functions. A holistic view on the regulatory mechanisms governing the dynamics of genes expression requires the understanding of the networking and interactions among genes and their products, since it is known that genes act in a collective manner to achieve biological functions [1,2]. By understanding the complex relations within these gene regulatory networks (GRN) we can highlight inhibitory or excitatory interactions, as well as how intracellular or extracellular factors affect gene products or dysregulate cellular processes.

The recent advances in biotechnology allowed simultaneous measurements of mRNA production from virtually all genes within genomes through high-throughput technologies such as the microarrays [3]. This resulted in massive amounts of data that proved to be a significant challenge for traditional analysis techniques due to the inherent combinatorial problems arising from the large number of variables. Advanced computational models are required to fill in the knowledge gap. These models have to cope with peculiarities of the data such as measurement noise. Additional challenges are posed by the modelling task, such as the under-determinism caused by the large number of variables compared to the available number of experiments. Also, since groups of genes are commonly acting to activate/inhibit a regulated gene, the computational search space grows to a much larger potential search space of virtually countless combinations [4].

Initial computational approaches for inferring gene regulatory networks from gene expression data consisted of classical techniques. Boolean networks are binary models that consider the expression of a gene to be either on or off, and model the effects of other genes on a specific target gene through a Boolean function. A major drawback is the fact they do not consider intermediate levels of gene expression, hence resulting in information loss [5-7]. Furthermore, they assume transitions between genes activation states to be synchronous, which is biologically implausible [8]. Bayesian networks (BN) are a mixture of probability calculus and graph theory, and attempt to model gene regulatory networks as directed acyclic graphs in which the nodes represent the genes and the edges connecting the nodes represent regulatory interactions, which are encoded via conditional dependences learned from the data. Although effective in dealing with noise, incompleteness and stochastic aspects of gene regulation, they fail to consider temporal dynamic aspects that are an important part of regulatory networks modelling [9]. Dynamic Bayesian networks (DBN) evolved feedback loops to effectively deal with the temporal aspects of regulatory networks but their benefits are hindered by the high computational cost required for learning the conditional dependencies in the cases where large numbers of genes are involved [10]. Linear additive regulation models [11] revealed certain linear relations in regulatory systems but failed to capture nonlinear dynamics aspects of genes regulation. Other approaches included differential equations, which besides the high computational costs have also the disadvantage of being sensitive to noisy data (as is the case of data resulting from microarray experiments) [2,12].

Recent approaches tried to overcome the drawback of traditional methods in several ways [13]. Keedwell and Narayanan [4] used a hybrid neuro-genetic algorithm to mitigate the under-determinism problem. Their method combines genetic algorithms (GA) with a single layer artificial neural-network (ANN), where each chromosome of the GA selects from the whole data set a small number of regulating genes and the ANN is used to determine how the expression levels of these input genes affect another gene's expression. However, the lack of a recurrent structure and the training method of the ANN may pose serious problems when modelling the complex temporal dynamics of gene expression regulation and renders the method vulnerable to local minima traps, respectively.

Some of the most effective approaches towards problems regarding temporal information processing are the recurrent neural networks (RNNs) [14] and recurrent fuzzy neural networks (RFNNs) [15]. Recurrent networks, in general, can deal with temporal and spatial/temporal problems by adapting self loops and backward connections to their topologies and structures, both of which are used to memorize past information. Recently, Xu *et al *[8] used an RNN combined with Particle Swarm Optimization (PSO) to capture the complex nonlinear dynamics of gene regulatory networks. Although RNNs are generally efficient for temporal sequence production problems, it has been shown that recurrent fuzzy networks outperform recurrent neural networks [16,17] in problems that involve concurrent spatial and temporal mapping like the one of regulatory networks reconstruction. Additionally, fuzzy-based approaches are better candidates in dealing with the uncertainties of modelling noisy data, due to their high-level, human-like reasoning [18,19]. In [18] regulatory interactions among genes are extracted in the form of fuzzy IF-THEN rules by searching for statistically significant fuzzy dependency relationships among genes. In [19], Sokhansanj *et al *perform an exhaustive search for possible rules describing gene interactions (they use the same IF-THEN formalism), under the framework of a linear fuzzy logic scheme that restricts the search space. However, both methods have the drawback of requiring prior data discretization, while [19] has the additional disadvantage of not considering temporal information.

In this study, we propose a novel multi-layer evolutionary trained neuro-fuzzy recurrent network (ENFRN) applied to the problem of GRN reconstruction, which addresses the major drawbacks of currently existing computational methods. Our choice was driven by the benefits, in terms of computational power, that neural network based methods provide [4,14,15]. The self-organized nature of ENFRN algorithm is able to produce an adaptive number of temporal fuzzy rules that describe the relationships between the input (regulating) genes and the output (regulated) gene. Related to that, another advantage of our approach is that it overcomes the need of prior data discretization, a characteristic of many computational methods which often leads to information loss [18-21]. The dynamic mapping capabilities emerging from the recurrent structure of ENFRN and the incorporation of fuzzy logic drive the construction of easily interpretable fuzzy rules of the form: 'IF gene *x *is highly expressed at time *t *THEN its dependent/target gene *y *will be lowly expressed at time *t*+1'. The evolutionary training, based on the PSO framework, tries to avoid the drawbacks of classical neural networks training algorithms. Additionally, we are approaching the under-determinism problem by selecting the most suitable set of regulatory genes via a time-effective procedure embedded in the construction phase of ENFRN. Also, besides determining the regulatory relations among genes, our method can determine the type of the regulation (activation or repression) and at the same time assign a score, which might be used as a measure of confidence in the retrieved regulation. Experiments on real data sets derived from microarray experiments on *Saccharomyces Cerevisiae *prove the ability of the proposed method to capture biologically established gene regulatory interactions, outperforming at the same time other computational methods.

## Results and Discussion

We applied our method on *Saccharomyces Cerevisiae *gene expression data obtained from cell cycle microarray experiments to identify regulatory interactions and construct putative regulatory networks. The cell cycle process consists of four phases: G1 (in which the cell grows and, under appropriate conditions, commits to division), S (in which the DNA is synthesized and chromosomes replicated), G2 (a 'gap' between S and M), and M (in which chromosomes are separated and the cell is divided). After the M phase, the cell enters the G1 phase, hence completing a 'cycle' [22].

The datasets we employed originate from a study containing measurements of virtually all yeast genes (6178) across 77 time points (experiments). In Spellman *et al*.'s experiments, yeast cell cultures were synchronized with different methods including *αlpha *factors arrest (two complete cycles, sampled each 7 min, totally 18 samples), temperature arrest of temperature sensitive mutants (*cdc15*, three complete cycles, sampled each 10 min, totally 24 samples) and *elutriation *synchronization (one complete cycle, sampled each 30 min, totally 14 samples) [23]. This data set includes also a subset from a previous experiment in which the cells were synchronized by temperature arrest of a *cdc28 *allele (17 samples taken at 10 min intervals) [24], as well as 4 samples initially intended for a study on cyclins CLN3p and CLB2p.

It must be noted at this point that all the results in this study were obtained using *cdc15 *as training set for the ENFRN models, while *alpha *and *cdc28 *subsets as testing data sets. The choice for this training set was based on the fact that it had the largest amount of time points. *Alpha *subset was chosen because it originates from the same experimental setting to the training data set and possesses the largest amount of data after *cdc15*. At the same time, it allows us to provide comparison to other methods that have used the same data set. *Cdc28 *was chosen to provide a means of testing the method against data originating from a different experimental setting than that of the training. For reasons of simplicity and coherence we will present in the following results based on *alpha *data set (used as testing set). Similar results were obtained when using *cdc28 *subset as testing set. Full details on the results of *cdc28*, as well as general settings of the parameters of the algorithm and additional information regarding both test data sets, are provided in 'Additional file 1'.

**Figure 1:**
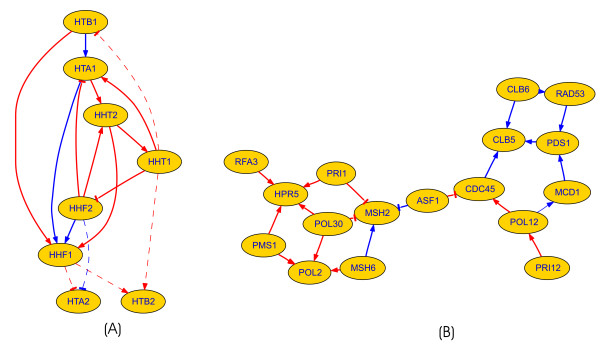
**Reconstructed small-size networks using the proposed ENFRN method**. The networks learned using our proposed method for (A) the 8 histones dataset and (B) using the third group of genes (out of the total 19 genes of the original set, RAD54 and POL1 are not shown as, there were found no interactions implicating them). Arrows correspond to positive and tee's to negative regulation. Edge colour reflects the composite score value as a measure of confidence in the interaction (blue corresponds to scores <0.5, red to those >0.5). Dashed lines correspond to retrieved interactions that have no genetic but physical interaction counterparts in experimental databases. The larger network (B) is characterized by more than 80% agreement with current biological knowledge.

To evaluate the performance of ENFRN and compare our results with those of other published methods, we used four groups of genes. The first one consisted of eight histone genes (HHT1, HHT2, HHF1, HHF2, HTA1, HTA2, HTB1 and HTB2) that encode the four core histones (H2A, H2B H3 and H4). The histones are the main protein components of chromatin, forming its fundamental packaging unit. Since the chromosomes (consisting among others of DNA and histones) need to be replicated before cell division, the expression of histone genes should be regulated tightly for the proper functioning of the replication process [23]. Using a composite score lower than 0.6 for selecting a regulatory interaction, we have built the network presented in Figure 1. Out of the 14 currently known genetic interactions we have retrieved 11. The rest of the interactions we have retrieved are indexed in the yeast databases as physical interactions, thus being indirectly supported (as shown in Table 1) [25,26]. The existence of a physical interaction among two entities reflects the fact that protein products of the respective genes interact at protein level and the existence of the interaction was experimentally proven. For example, the HHF1 - HTA2 was marked as a physical interaction by several Affinity Capture-MS experiments [27]. This could reflect the existence of certain correlations in their gene expression level, therefore triggering our method to retrieve it as a valid interaction. Table 1 lists all extracted interactions, their type and corresponding composite score, as well as comparative information on the currently existing biological knowledge. It should be noted that when comparing to the results of Chen *et al *[28], our method retrieved 16 interactions (both genetic and physical), while only 12 were retrieved with the Bayesian Network approach of Chen *et al*. The comparison is even more relevant when restricted to the genetic interactions, which are the main goal of the compared approaches (due to the gene expression data employed): ENFRN-based method identified 11 genetic interactions out of 16, as noted above, while in the case of the Bayesian Network only 6 out of 12 were genetic interactions. Table 2 provides intermediate results regarding the ENFRN performance during the optimization process.

**Table 1:** Information regarding the extracted interactions based on *alpha *test data set.

a/a	Regulator	Target	Type	Composite Score	Genetic Interaction	Physical Interaction
1.	HTB1	HTA1	+	0.49589	1	1

2.	HTA1	HHF1	+	0.44116	1	1

3.	HHF1	HTA2	-	0.58690	0	1

4.	HTB1	HHF1	+	0.59115	1	1

5.	HHF1	HTB2	+	0.53736	0	1

6.	HHF2	HTA1	-	0.54367	1	1

7.	HHF2	HTA2	-	0.46783	0	1

8.	HHF2	HHF1	+	0.48136	1	1

9.	HHT1	HTA1	+	0.56520	1	1

10.	HHT1	HTB1	-	0.59864	0	1

11.	HHT1	HTB2	+	0.52335	0	1

12.	HHT1	HHF2	-	0.59846	1	1

13.	HTA1	HHT2	+	0.58984	1	1

14.	HHT2	HHF1	+	0.52567	1	1

15.	HHF2	HHT2	+	0.53911	1	1

16.	HHT2	HHT1	+	0.55456	1	1

The table shows regulator and target genes implicated in the interaction, the type of regulation determined and the value of composite score. In the last two columns we provide the kind of biological evidence that supports the interaction.

**Table 2:** Information regarding the increase of efficiency and simultaneous decrease of complexity throughout the various training phases of ENFRN

			Composite Score Values in ENFRN Structures			Number of Rules and Output Nodes in ENFRN Structures			
a/a	Regulator	Target				Initial		Simplified	
			
			Initial	Simplified	Trained	Rules	Output	Rules	Output
1	HTB1	HTA1	0.899	0.935	0.533	12	9	12	9

2	HTA1	HHF1	1.056	0.988	0.435	11	8	9	8

3	HHF1	HTA2	0.644	0.698	0.595	12	10	11	10

4	HTB1	HHF1	0.842	0.815	0.536	12	8	10	8

5	HHF1	HTB2	0.654	0.642	0.560	12	8	10	8

6	HHF2	HTA1	0.741	0.773	0.618	14	10	12	10

7	HHF2	HTA2	0.657	0.669	0.543	14	10	12	10

8	HHF2	HHF1	0.748	0.785	0.503	14	9	12	8

9	HHT1	HTA1	0.898	0.916	0.664	12	9	10	9

10	HHT1	HTB1	1.095	1.097	0.811	12	10	10	9

11	HHT1	HTB2	0.632	0.660	0.567	12	8	10	8

12	HHT1	HHF2	0.841	0.791	0.755	12	9	10	9

13	HTA1	HHT2	1.233	1.188	0.606	11	9	8	8

14	HHT2	HHF1	0.658	0.625	0.567	14	9	14	9

15	HHF2	HHT2	0.890	0.881	0.589	14	10	13	10

16	HHT2	HHT1	0.962	0.975	0.749	14	10	10	9

We can depict a decrease at the complexity levels (i.e. 87% of the 16 extracted interactions described in table) between the initial and the simplified structures followed by a corresponding decrease in the score levels between the phases of the initial and the trained ENFRN.

**Figure 2:**
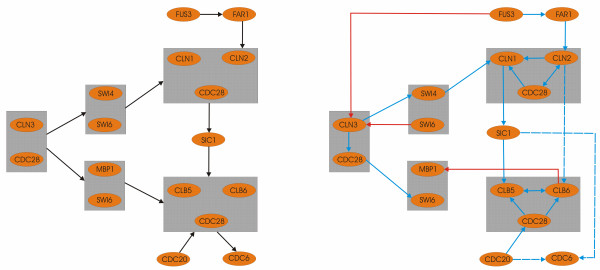
**Reconstruction of a KEGG pathway**. In this figure we present the KEGG pathway (A) and the reconstructed analogue of our ENFRN-based method. A grey shaded rectangle represents genes that compose a complex. The edges inside these circles are considered as correct edges since genes inside the same circle will co-express with some delay. In blue colour we have correct determined interactions, in dotted blue we have interactions that are true via an intermediate gene and false interactions are indicated in red colour. 71.4% of the interactions extracted were in absolute accordance with biological knowledge, and this percentage rose to 85.7% when considering the interactions that were correct via an intermediate gene.

In a second experiment we further tested and compared our approach against other well known algorithms for GRN reconstruction, on a well studied pathway consisting of 14 genes. Specifically, we have compared against Bayesian Networks (BNs) and Dynamic Bayesian Networks (DBNs). The target sub-network we studied (part of a SS cell cycle pathway consisting of 45 genes around the cyclin-dependent protein kinase CDC28/YBR160w) has been used as a benchmark dataset in many similar studies [10,13]. Our method outperformed the ones we have used for comparison. As we can resolve by inspecting Figure 2 and Table 3, our ENFRN-based approach extracted 15 biologically validated interactions (when BN had 3 and DBN had 5), 4 interactions that are biologically validated via an intermediate gene (when BN had 4 and DBN 4) and 3 interactions that are erroneous (when BN had 7 and DBN 4). Additionally, in order to explicitly test the efficiency of our proposed hybrid network, we have replaced ENFRN with another neuro-fuzzy recurrent network named RSONFIN [16], and applied it within the same framework for reconstructing GRNs described in this paper. The results shown in 'Additional file 1', indicate the superiority of ENFRN in terms of both biologically validated interactions and computational time. ENFRN managed to infer the reconstructed GRN faster than all methods used for comparison. Additional details and evidence regarding the computational times of the compared methods are provided in 'Additional file 1'.

**Table 3:** This table represents interactions detected by the three computational models, ENFRN, BN and DBN.

					Model Derived Interaction		
					
a/a	Regulator	Target	Composite Score	Type	ENFRN	DBN	BN
1.	FUS3	FAR1	0.35898	T	√	-	-

2.	FAR1	CLN2	0.35138	T	√	-	-

3.	SIC1	CLB5	0.53303	T	√	-	-

4.	CLB5	CLB6	0.40858	T	√	-	-

5.	CLB6	CLB5	0.53303	T	√	-	√

6.	CLN2	CLN1	0.54337	T	√	-	√

7.	CLN3	CDC28	0.34730	T	√	-	-

8.	CLN1	SIC1	0.54336	T	√	-	√

9.	CDC28	CLB6	0.41569	T	√	-	-

10.	CLB6	CDC28	0.39661	T	√	-	-

11.	CDC28	CLN1	0.54336	T	√	-	-

12.	CLN3	SWI4	0.33336	T	√	√	-

13.	CDC20	CDC28	0.49565	T	√	-	-

14.	CDC28	CLB5	0.53309	T	√	-	-

15.	SWI4	CLN1	0.38757	T	√	√	-

16.	CLN2	SIC1	-	T	-	√	-

17.	SIC1	CLN2	-	T	-	√	-

18.	CLN1	CLN2	-	T	-	√	-

19.	CLN2	CLB6	0.38983	I	√	√	-

20.	CDC20	CDC6	0.40848	I	√	√	√

21.	SIC1	CDC6	0.55806	I	√	-	-

22.	FAR1	SIC1	-	I	-	-	-

23.	FAR1	FUS3	-	I	-	√	√

24.	FUS3	CDC28	-	I	-	√	

25.	CLN1	FAR1	-	I	-	-	√

26.	CLN1	CLB5	-	I	-	-	√

27.	CLB6	MBP1	0.37033	F	√	-	-

28.	SWI6	CLN3	0.56508	F	√	-	-

29.	FUS3	CLN3	0.57180	F	√	-	-

30.	CDC6	SIC1	-	F	-	√	-

31.	CDC6	CLB6	-	F	-	√	-

32.	CDC6	CLB5	-	F	-	√	-

33.	CDC20	FAR1	-	F	-	√	-

34.	SIC1	FAR1	-	F	-	-	√

35.	CDC6	FUS3	-	F	-	-	√

36.	FAR1	CLN3	-	F	-	-	√

37.	CLB5	MBP1	-	F	-	-	√

38.	SIC1	MBP1	-	F	-	-	√

39.	MBP1	SIC1	-	F	-	-	√

40.	CDC6	CLN3	-	F	-	-	√

The 3^rd ^column provides scores of the final trained ENFRN structure responsible for determining a specific interaction. Column 'Type' indicates whether a determined interaction is true (T), true via an intermediate gene (I) or false (F). In the last column we indicate which models detected the interaction described in columns 1 and 2.

We next proceed evaluating our approach on larger datasets. Hence, the third set we tested, consisted of 19 genes which include DNA polymerases (POL1, POL2, POL12, and POL30), DNA helicase (HPR5), type B cyclin genes (CLB5 and CLB6), DNA primases (PRI1 and PRI2), radiation sensitive genes (RAD53 and RAD54), repair related genes (MSH2, MSH6, and PMS1), replication protein A encoding gene (RFA3), DNA replication initiation factor (CDC45), securin gene (PDS1), nucleosome assembly factor (ASF1), and a subunit of the cohesin complex (MCD1). These genes play an important role in the process of cell cycle conducting important cellular activities such as DNA replication initiation, DNA damage-induced checkpoint arrest, DNA damage repair, formation of mitotic spindle, and so on. As observed in Figure 2, ENFRN retrieved 21 regulatory interactions among 17 genes (there was no interaction found for RAD54 and POL1). Bold edges denote retrieved interactions that are backed by genetic interaction while dashed edges correspond to those having physical interaction counterparts in experimental databases (Biogrid and SGD). Thin edges denote interactions that could not be found neither in Biogrid nor SGD. For 16 out of 21 interactions there exists biological experimental evidence stored in the Biogrid and Saccharomyces Genome Database (SGD). This is more than double the number of valid interactions the method of Chen [29] found using the same group of genes. Of particular interest is the upregulation of cyclin CLB5 involved in DNA replication during S phase by the securing gene PDS1. This regulation is substantially supported by experimental evidence at both genetic and protein level [30,31], however it has not been retrieved by Chen *et al*'s method. The composite score of ENFRN for this interaction was relatively low (0.36518), thus underlining the high confidence of the retrieved regulation. An interesting case is that of PRI2-POL12, which we inferred as a valid interaction. Although we could not find existing biological experimental information regarding the respective genetic interaction, there was significant evidence of physical interaction among the protein products of this pair of genes from a series of Affinity Capture-MS experiments [32,33]. It must be noted that this interaction was marked as a valid genetic interaction by Chen *et al *[28], although their cited evidence highlighted it as a protein-protein interaction via Tandem Affinity Purification experiments [32,34]. A complete list of our interactions, their composite scores and experimental information can be found in 'Additional file 1'.

**Figure 3:**
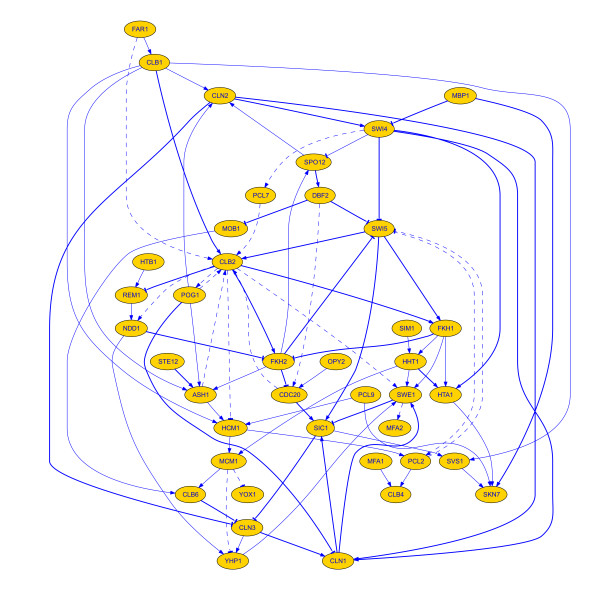
**Medium-size reconstructed network**. Gene network retrieved using the group of 41 genes (properties of edges follow the ones described in Figure 1). No regulatory interaction was found for MNN1. The 32 genetic interactions, 16 physical interactions and 8 intermediate connections correspond to 67.4% accordance with current biological knowledge. All interactions have a composite score below 0.5 and therefore edges are blue coloured.

In a final experiment we aimed at testing the performance of ENFRN on a larger group of genes. This group of genes was selected by Chen *et al *and includes 20 cell-cycle target genes and their regulators [29], a total of 41 genes. Due to the particularities of this dataset (e.g. the cyclin CLB is a densely connected hub-like gene [25]), we have relaxed the constraint on the number of possible regulators for a gene from 5 to 8. As presented in Figure 3, our method retrieved a total of 83 interactions. Out of these, 32 were found to have corresponding genetic interaction experimental evidence, while 16 interactions corresponded to previously proven physical interactions [25,26]. This totalled to 57.8% accordance to currently valid biological knowledge. Interestingly, we have found 8 cases in which experimentally valid interactions from the Biogrid database are connected in our network through only one intermediate node. As noted by other studies, these indirect interactions might reflect a conjunctive action of the respective entities for the regulation of the target entity [28,29]. This means that a total of 67.4% of the edges in our network are supported directly or indirectly by biological experimental evidence.

Our results highlight the crucial role of cyclins in the cell cycle, of which the most typical is CLB2 as reported by others [29,35]. Accordingly, ENFRN identified the upregulating interaction among CLB1 and CLB2 [29]. Worth to be noted is also the activation of the NDD1 by the CLB2 that takes place in the G2 phase of the cycle, which is part of the larger scale interactions between CLB2 and the SFF complex (consisting of FKH1, FKH2 and NDD1-directly connected in our network) [29,35]. MCM1, a DNA binding protein that works with the SFF complex, has been placed in direct interaction with FKH2, thus highlighting its role within the complex.

The important role of the cyclin-dependent kinase SIC1 is captured by its close interaction with the G1-cyclins CLN1,3. Additionally, the documented inhibition of SIC1 by the SFF component FKH2 and the inhibition of SWI5 by the same FKH2 were correctly retrieved [29,36]. The control of the cyclins CLN1,2 by the SBF complex (via SWI4 activation of CLN1) [37,38] is reflected by the direct interconnection among these genes. Also, the suggested upstream activation of CLN1 by CLN3 at the beginning of the cell cycle [39] was promptly retrieved by our method.

In a number of cases the microarray data show transcriptional co-regulation patterns and although the gene products are not physically interacting, this tight genetic connection might suggest that genes sustain indirect connections through other gene products [40]. Computational methods usually accommodate this scenario by considering indirect connections through one or even two intermediate nodes [28,29,40]. A large number of such interactions can be identified in our network, such as the indirect activation of CLB2 by the SBF and MBF complexes (SWI4 and MBP1, through PCL7) [35]. Another example is the loop where the transcription of SWE1 activated by CLN3 takes place in our network indirectly, through genes CLN1 and YHP1 [29]. Additional information on the interactions and their composite scores is presented in 'Additional file 1'.

As a final comment we should notice that many of the gene regulatory interactions take place exclusively at a specific phase of the cell-cycle, the pair of interacting genes remaining silent for the rest of the cycle. Problems arise in such cases from the reduced availability of experimental samples (e.g. G2 phase of the cycle is represented in the experimental dataset we used by only 2 samples). Despite the great difficulties deriving from this fact, we have proven that the proposed ENFRN-based method managed to acquire biologically validated relations among genes from about 60% to even 100% (depending on the data set) percent of the extracted interactions.

## Conclusions

Fundamental processes occurring in organisms are carried out through complex networks of regulatory interactions among genes and their products. The inference of gene regulatory networks based on experimental data obtained from microarrays has become an important way to understand these regulatory mechanisms. Herein we describe a novel evolutionary trained neuro-fuzzy recurrent network to model the regulatory networks and reveal interactions between genes. Our model is able to identify potential regulators of genes through a time efficient process. The recurrent structure of ENFRN ensures that the dynamics of gene expression are properly retained while, at the same time, the self-organizing properties of the method automatically account for the discretization process. The self-organization combined with the inherent fuzzy logic successfully manage to deal with the important amount of noise present in microarray data and at the same time provide an efficient representation of the interactions through fuzzy rules.

One of the key characteristics of the proposed approach is that by incorporating ENFRNs neural network-specific processing and learning capabilities the method can be applied (after the training process is concluded) to unseen samples arriving from microarray data of different experimental settings in order to test whether the same regulatory interactions are valid within the new data set.

The proposed ENFRN-based method successfully extracted relations that in their majority were in accordance with biologically proven regulatory interactions, outperforming other computational approaches. In certain cases our algorithm picked up interactions that could not be retrieved in the lists of experimental interactions currently existing in databases. It might be possible that some of these interactions are valid but currently unknown, since the available experimental data is still far from complete at present. However, those interactions and especially the ones assigned a high degree of confidence should be further examined.

## Methods

### Evolutionary Recurrent Neuro Fuzzy Network

This section presents the proposed method, its architectural structure together with key aspects of its functioning: dynamic mapping capability, temporal information storage and fuzzy inference system.

Unlike other neuro-fuzzy network architectures, where the network structure is fixed and the rules should be assigned a priori, there are no fuzzy rules initially in the architecture we are presenting; they are constructed during learning, in a self organized manner. The two learning phases (the structure and parameter learning) are used to accomplish this task. The structure learning phase is responsible for the generation of fuzzy IF-THEN rules as well as the judgment of the feedback configuration, and the parameter learning phase for tuning the free parameters of each dynamic rule (such as the shapes and positions of membership functions). These are accomplished through repeated training on the input-output patterns. The way the input space is partitioned determines the number of rules.

Under the framework of the GRNs reconstruction that this paper addresses, the input and output spaces correspond to the expression profiles of the input and output genes, respectively. A fuzzy rule derived by ENFRN is time-dependent and is realized via linguistic labels corresponding to fuzzy sets (represented in this study by Gaussian membership functions) that describe the variations of the expression profiles of the input-output genes. Those labels are unified in a rule via a fuzzy AND operation:

### IF *g*_1_(*t*-1) is low AND *g*_2_(*t*-1) is high AND ... *g*_*N*_(*t*-1) is low THEN *g*_*o*_(*t*) is high

where low, medium, and high correspond to linguistic labels represented by fuzzy sets and partition the input and output space. The left hand side of a rule such as the one described above corresponds to a certain cluster of the expression profiles of the input genes (regulators), while the right hand side on a corresponding partition of the output space (target genes).

Given the scale and complexity of the data, the number of possible rules describing the gene expression data at hand is kept under constraint by employing an aligned clustering-based partition method for the input space, meaning that both input and output variables may have a different number of fuzzy sets describing them [16,41]. Additionally, we enforce a setting in which rules with different preconditions may have the same consequent part. By incorporating the clustering scheme, we are able to tackle the problem of rule set combinatorial explosion that appears when using other neuro-fuzzy approaches [42].

### ENFRN Architecture

**Figure 4:**
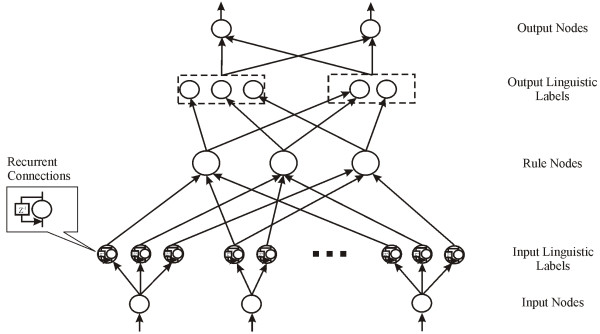
**Architectural structure of ENFRN**. Some of the basic properties of the network are the ability to allow each input and output variable to be described by a different number of linguistic labels and that more than one rule can be assigned the same antecedent.

Following, we give a detailed layer by layer description of the multilayer architectural structure of ENFRN described in Figure 4. The purpose of the following description is to indicate the signal propagation and the operation functions of the nodes in each layer. Throughout the paper, we use the symbol *φ*_*i *_^(*k*) ^to denote the input of node *i *in the *k*-th layer while the symbol *ψ*_*i *_^(*k*) ^will denote the output of node *i *in the k-th layer.

**Layer 1**: Every node in the first layer represents an input variable in the ENFRN. The values of the nodes on this layer are directly transmitted to the next layer without any computation.(1)

where *x*_*i *_is the value of the *i-*th input (gene expression) value.

**Layer 2**: Each one of the nodes in this layer represents a Gaussian membership function that corresponds to a linguistic label (e.g. low-expressed, average-expressed, highly-expressed, etc.) and is described by:(2)

where *c*_*ij *_and *σ*_*ij *_corresponds to the mean and standard deviation of the Gaussian membership function, respectively and *ψ*_*ij *_is the *j*-th membership function of the *i*-th input variable. Additionally, in this layer we have the implementation of recurrence in ENFRN, specifically the input for each one of the nodes in this layer at a certain time point *t*, is described by:(3)

where *β*_*ij *_denotes the link weight for the feedback unit and *φ*_*ij *_is the *j*-th membership function of the *i*-th input variable. As it can be deduced from (3) each linguistic node, acts also as a memory unit storing past information for ENFRN concerning the input.

**3^rd^Layer**: The third layer, named Rules Layer, is comprised of rule nodes. Each one of the rule nodes performs precondition matching for the corresponding rule, using the following chain of fuzzy AND operations to incorporate its input values:(4)

where:(5)

where *D*_*i *_= diag(1/*σ*_*i*1_, 1/*σ*_*i*2_, ..., 1/*σ*_*in*_), *c*_*i *_= (*c*_*i*1_, *c*_*i*2_, ..., *c*_*in*_)^T^. The output of every one of the rule nodes corresponds to the firing strength of a rule; meaning how well fitted is the corresponding rule, such as to adequately describe the current input pattern.

**4^th^Layer**: ENFRN partitions the output space. The nodes are called output linguistic nodes and correspond to the subsequent part of a fuzzy rule; the output for every node is given by:(6)

As can be deduced from the equation above, the output for every one of the nodes of the current layer is the sum of the output of the rule nodes that have as consequent part the current node.

**5^th^Layer**: In this last layer we have the defuzzification process:(7)

*w*_*ij *_are the widths of the fuzzy sets of the output layer. Each one of the nodes corresponds to the value that ENFRN has calculated to be the predicted output value for the corresponding output variable.

### Partitioning of input and output space

The creation of a new rule within ENFRN corresponds to the creation of a new cluster in the input space. Therefore the way the input space is partitioned (clustered determines the number of fuzzy rules created. Thus, the number of rules created by ENFRN is problem depended, the more complex a problem is, the greater becomes the number of rules.

In order for the ENFRN to decide whether a new rule must be generated for the description of an incoming pattern (**x**, **y**), a two criteria scheme is adapted. The first one computes the overall error *E*_*k*_, which is defined as the difference between the output of ENFRN and the input signal and is given by:(8)

where *y*_*k *_is the value computed by the model based on the current input pattern *x*_*k *_while *y*_*k *_^*d *^is the desired output for the same pattern.

The second criterion is based on the calculation of the distance *d*_*i *_between the observation *x*_*k *_and all the existing till point rules(9)

where *N*_*r *_is the number of the rules, each one of the *d*_*k *_is computed by using equation (5). Then we find the(10)

If both the criteria below are true then, we create a new rule(11)

The first criterion checks whether the error of the model is greater than a specific value, indicating that under the current state of the network we cannot handle the new pattern without a large value of error. The second checks to see whether the pattern is 'close' enough to a cluster, so that it can become a member of it, or if a new cluster has to be created for it.

If the procedure described leads to the creation of a rule, the next step is the assignment of initial centers and widths of the corresponding membership functions. Since later on (at the structure fine tuning phase of the learning algorithm) the ENFRN structure will be optimized, we simply set:(12)

where *δ *is a constant deciding the overlap of the clusters, *d*_*min *_is the distance of the closest cluster (from the current pattern), and *c*_*i*_, *σ*_*i *_are the center and width for the membership function of each input variable. Similar methods have been used before, such as [41,43]. To reduce the number of fuzzy sets of each input variable and to avoid the existence of redundant fuzzy sets, we check the similarities between them in each input dimension. For the similarity measure of two fuzzy sets we use the formula previously derived in [44], which concerns bell - shaped membership functions.

Finally, ENFRN has to determine if a new output cluster must be created. A cluster in the output space is the consequent of a rule. We have already mentioned that one of the characteristics of ENFRN is that more than one rule can be connected to the same consequent. As a result, the creation of a cluster in the input space does not necessarily mean a subsequent creation of a cluster in the output space. It depends on the incoming pattern, since the newly created cluster in the input space could be connected to an already existing cluster of the output space. The model decides whether or not to create a new output cluster based on the two criteria described above (Eq. 11).

### PSO for ENFRN Optimization and Learning

**Figure 5:**
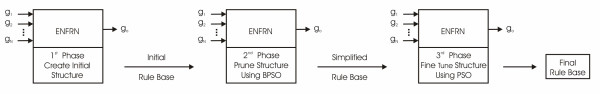
**ENFRN learning phases**. Overview of the three phases of ENFRN training cycle. The first phase represents the initial creation of the ENFRN structure, in the second phase the structure is simplified by pruning the rules obtained in the first phase, while in the third phase the fine-tuning of the ENFRN parameters is performed.

After the creation of the initial structure described above, ENFRN enters a two phase learning process where the initial structure is optimized and the various parameters (e.g. centers and widths of the fuzzy sets as well as link weights among connections) are fine tuned (as can be visualized in Figure 5). Both phases of this process are based on PSO.

PSO is an algorithm that simulates the social behaviour of organisms, such as birds in a flock [45]. This behaviour can be described as an automatically and iteratively updated system. In PSO, a particle represents a potential solution and its location or position in the search space is represented by a vector **X**_*i *_= (*x*_1_, *x*_2_, ..., *x*_*M*_). The swarm of particles moves through the problem space with a velocity that is represented for every particle by a vector **V**_*i*_. At every iteration PSO provides a quality measure for the current position (candidate solution) of each particle, via a function called fitness function (FF), in which **X**_*i *_is used as input. Each particle makes use of its own memory to keep track of its own best position *B*_*i *_(based on the FF value) attained so far. Additionally every particle has access to the best position among all particles *B*^*G*^. The new velocity for the next iteration is computed for every particle based on the particle's own best position as well as the general best position, like:(14)

where *c*_1 _and *c*_2 _are positive constants and *n*_1 _and *n*_2 _are uniformly distributed random numbers between 0 and 1. While there are various different neighbouring schemes for different problems, in this study we follow the standard version of PSO, where each one of the particles follows the best position acquired by the flock as can be depicted by Eq (14). These new velocities are then used to compute the particles new position, according to:(15)

Particles move through the problem space following a current of optimum particles using Eqs. (14), (15). The process is then iterated a fixed number of times or until a predetermined minimum error is achieved where hopefully, given a sufficient number of particles the swarm will converge towards an optimized solution [45].

### Structure Optimization

As we have already mentioned, during the creation of the initial ENFRN structure we do not spent much time determining the centers and widths of fuzzy sets so as to find a perfect cluster. This might drive ENFRN, especially in cases where we have difficult problems such as the one we study here, to create a rather large structure containing many rules that could even be redundant.

Hence, at this phase of the learning algorithm of ENFRN we have developed a scheme for deleting some of the rules along with their corresponding output nodes (in cases where they are not connected to other rule nodes), if they are either redundant or the patterns they describe could be efficiently represented by other rules. Therefore, the objective of this part of the learning process has a dual goal of decreasing the redundancy and simplifying the model. For the optimization of the ENFRN structure we have employed a discrete version of the PSO algorithm.

To optimize the number of rule nodes in the initial network structure we use a version of Binary PSO (BPSO) algorithm. Potential solutions to our optimization problem are encoded through particles that under BPSO are symbolized as fixed size binary strings *X*_*i *_= (*X*_*i*1_, *X*_*i*2_, ..., *X*_*iM*_), where *X*_*ij *_∈ {0,1}. Given a list of rule nodes, **R **= (*R*_1_, *R*_2_, ..., *R*_*M*_) the first element of **X**_*i*_, *X*_*i*1 _corresponds to the first rule *R*_1_, the second to the second rule *R*_2 _and so forth. A value of zero at the coordinate associated to a rule node, indicates that the corresponding rule is not selected, while unity indicates that the rule is selected.

It is apparent that the fitness function should be selected against the number of rule nodes as well as the performance of the network. The fitness function we are using in this paper for the BPSO is described as(16)

where MSE is the well known mean square error, *M *is the initial number of rules, as given by the structure learning phase and *N *is the number of rule nodes present in the simplified structure. We can derive from the formulation of the problem that we assume the number of rule nodes described by each one of the particles to be lower or at the most the same with the initial number of rule nodes (therefore it is 0<*N*<*M *→ 0 <*M*-*N *→ *M*-*N*>0 → *M*-*N*+1>1). It is obvious from equation (16) that the closer a particle arrives to an optimized solution in terms of small value of the error with a minimum number of rule nodes, the smaller the fitness value is obtained. Hence, BPSO attempts to minimize the function (16).

The initial population or swarm of particles is composed of *a certain number of *randomly generated binary strings. Every coordinate (position) of *X*_*ij *_of a particle is a uniform random number *r *drawn on the interval (0,1). In BPSO as in PSO, every one of the particles is associated with a unique vector **V**_*i *_= (*Vi*_1_, *Vi*_2_, ..., *V*_*iM*_). The members *V*_*ij *_in **V**_*i *_establish the rate of change for each one of the corresponding coordinate *X*_*ij *_in **X**_*i*_. Each element *V*_*ij *_in **V**_*i *_is updated according to the equation:(17)

Where the parameters *c*_1_, c_2_, *n*_1_, *n*_2 _and *W*_*I *_are the same as the one for equation (14) of PSO.

As we have already mentioned, the value for every coordinate of the *i*-th particle **X**_*i *_can be either 0 or 1. The algorithm decides on the value based on its respective velocity *V*_*ij *_and is given by(18)

Where *n*_3 _is a uniform random number in the range [0,1], and(19)

that is the well known sigmoid function.

### Parameters Learning

In this section we describe the algorithmic methodology we follow to train the parameters of the ENFRN. Several variations of the family of back propagation algorithms (e.g. Back Propagation through time) have been used to train recurrent multilayer neural and fuzzy neural networks by means of error propagation via variational calculus [46]. Even though they are widely used, these algorithms have many drawbacks. For instance, their success depends upon the quality of the training data, as well as the initial values of the weights of the network. Additionally, they are known to be easily getting trapped in local minima, losing this way the global minimum of the error function [47].

The key idea of using PSO in this final phase of the training process is to fine-tune the centres and widths for the fuzzy sets comprising the rule and output nodes, as well as the weights assigned to the recurrent links, so as to have the minimum number of errors in the prediction of the model. Under the PSO formalism, the first step towards the training of ENFRN is to create a number of particles, each of which will be represented by a vector like:(20)

where *k *is the number of input variables, *N*_*o *_is the number of output nodes, *m*_*ij *_and *σ*_*ij *_represent the centers and widths of the fuzzy sets describing the input variables, *β*_*r *_corresponds to the centers of the fuzzy sets of the output variable, while *w*_*ij *_are the recurrent weights of the 2^nd ^layer of ENFRN.

A large number of different fitness functions can be used for the training process, here we are using the following:(21)

where *T *is the number of time points in the dataset, *y*_*i *_represents prediction of the output based on input value as calculated ENFRN and *y*_*i *_^*d *^is the actual value given by the dataset. Hence the purpose of applying PSO in this final phase of ENFRN learning scheme is to minimize (21) by fine-tuning the parameters of ENFRN structure.

### ENFRN reconstructs GRNs

In this section we present the approach through which, given an ENFRN structure trained and optimized as described above, we can determine if the input regulates the output, the kind of the regulation and also provide a quantitative measure (which we call regulation score) specifying how well this task is performed. Additionally, we propose our approach on handling the important and computationally expensive problem of determining a set of possible regulators for a certain target gene.

### From ENFRN Structure to Regulation Type

A set of fuzzy rules arriving from a certain ENFRN structure is employed to determine the regulation type (i.e. up, down, or even no regulation) that the input imposes to the output based on a given data set. Each of the ENFRN rules has a prerequisite part consisting of a number of fuzzy sets (matching the number of input variables). These fuzzy sets correspond to linguistic labels describing the values of the input variable. In our case the linguistic labels correspond to a certain expression level of the input gene (e.g. high, medium-high, medium, etc). The same applies for the output.

Throughout this study, we follow the main principle that for a certain gene *x *to be considered as a possible regulator of some other gene *y*, then *x *must have a previous expression status alteration when compared to *y*. An expression status alteration (both for input and output genes) occurs when the linguistic label describing the expression status of a gene at a specific time point *t *is different than the value of the linguistic label of the same gene at time point *t*-1.

We can mathematically formulate the proposed framework using a function that will provide a Regulation Score (RS) in a given ENFRN structure:(22)

with(23)

where *x*_*i *_^*t *^and *y*_*i *_^*t *^are the linguistic labels describing the input and output at time point *t*. *T *is the number of time steps present at a certain dataset, *N*_*v *_is the number of the input variables, *N*_*IC *_is the number of times that input(s) change(s) expression value between consecutive time points, and *f *is specified as a Kronecker delta function, i.e.:(24)

As it can be deduced from (22) and (23) we follow a first order Markov process where we search for an alteration at the expression levels of all input and output variables/genes between 2 consecutive time points. However, due to the recurrent nature of the ENFRN, the proposed approach actually takes under consideration all of the previous time dependencies during the construction and learning processing phases.

**Figure 6:**
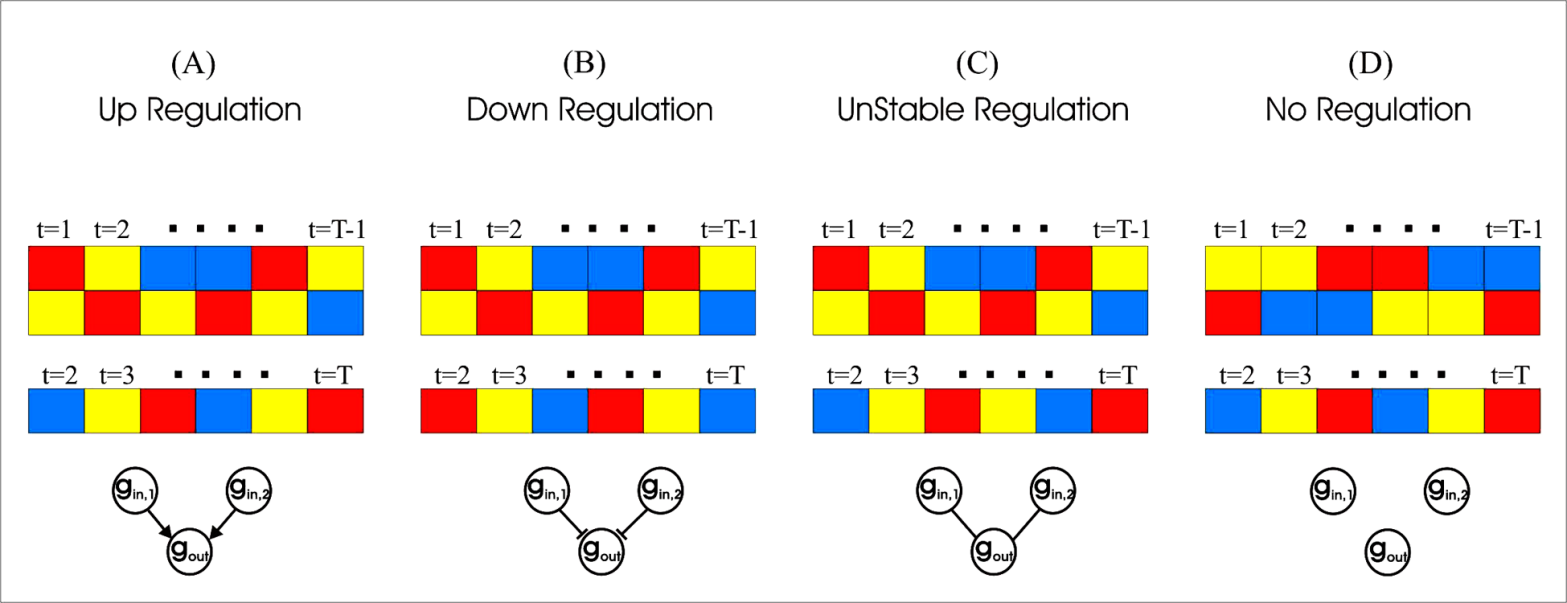
**Linguistic rules to regulation type**. Simplistic representation of the methodology we follow to translate a set of fuzzy rules extracted by an ENFRN model to the type of regulation (if any) input gene(s) impose to the output gene. As deduced from figures, the type of regulation (A) up, (B) down, (C) none is determined by the output given that there is an earlier alteration at the expression of the input, without which we can not deduce regulation (d).

The output of (22) can be either positive, negative or zero, corresponding to up, down and no regulation respectively (Figure 6, A-C). Inspecting formulas (22), (23) and (24) we can deduce that the output variable is mainly responsible for determining the type of regulation, given that there is a corresponding prior (in terms of time) alteration of expression status in the input variables. Indeed, if there is a decrease in the expression levels of the output gene/variable from time point *t *to time point *t*+1 then the term (*y*_*t*+1_-*y*_*t*_) becomes positive, otherwise it is negative. On the other hand the term (*x*_*t*+1_-*x*_*t*_), which through (24) gets a discrete value of 0 or 1, solely indicates whether or not there is a prior change in the expression of the input. The sign of RS determines whether the output is up or down regulated by the input. As we can observe from (22), (23), we normalize RS, by diving it with the number of times the input changes expression value. The value of the proposed score lies between 0 and 1 with 0 being the best case, i.e. the number of times the input gene changes expression values is followed by an equal number of alterations in the expression values of the output. Unstable regulation (RS = 0) means that the input cannot provide a standard type of regulation for the output or, in other words, for half of the cases where we have an alteration of the expressional status of the input we have up-regulation, while for the other half we have down regulation.

If there is more than one input variable in the Input Layer then those input variables/genes are connected thought a fuzzy AND operation at the Rule Layer. Hence, all of the input genes must have a simultaneous change in their expression profiles. Therefore, in the case of multiple inputs, if the alteration of the input variables is not simultaneous for any time point of the experiment, the output of RS indicates that the input genes do not regulate the target (Figure 6-D).

### Determining potential regulators

Irrespective of the algorithmic methodology followed for determining the possible regulation of a set of genes to some target gene(s), the major problem for gene networks reconstruction is the computational complexity rising from the enormous number of candidate regulators (i.e. feature space) that should be considered. This fact makes the application of many data-driven computational models prohibitive for accurate prediction of large scale modelling of regulatory networks. Various tactics [4,8,48] have been used to overcome this significant problem. One of those techniques is to apply clustering [48] towards the construction of co-expressed clusters of genes, then further analysis proceeds using just the centres (i.e. mean) of those clusters. This approach however imposes a very serious amount of information loss since only general trends concerning groups of genes can be inferred and not real biological associations among pairs of genes.

Another approach is to employ heuristic or evolutionary schemes, like PSO, for the determination of the best regulators for a certain target gene [8]. Specifically, starting from some gene population, BPSO feeds randomly various input subsets of the initial population to the main method used (e.g. ANN) to determine the regulation. Finally, BPSO concludes on the best regulators using as fitness function value the output of the main method [4]. The application of such algorithms at this point of the GRNs reconstruction actually leaves the problem intact. Indeed, considering as input to those algorithms large set of genes would induce severe computational complexity. The problem is magnified if we consider that based on the stochastic nature of those algorithms the whole process must be repeated several times.

**Figure 7:**
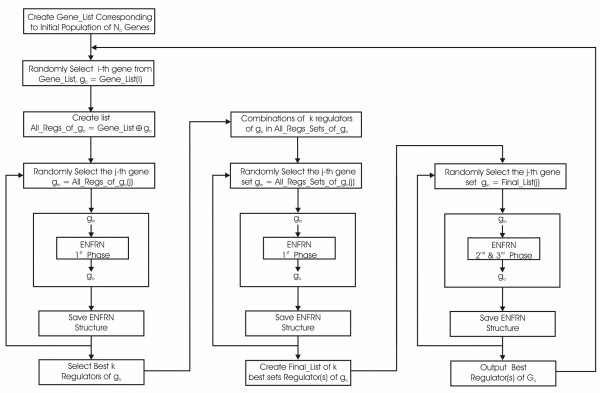
**General framework for GRN reconstruction**. Detailed schematic description of the three-stage process we propose for determining the regulators of each one of an initial population of *No *genes. As we can depict in the first stage of the process (column 1) *N*-1 ENFRN structures are created for each one of the *N *genes. Sequentially combinations of the best *N*_*B *_regulators are tested for the output gene. Finally using phases 2 and 3 of the ENFRN, we fully train the ENFRN networks (column 3) the best sets of regulators for each one of the output (target) genes.

Within the methodology we follow to reconstruct GRNs we have developed a procedure that identifies sets of potential gene regulators by making use of the ENFRN resources. The key idea of the procedure we follow (visualized in Figure 7), is to take advantage of the computational efficient first phase of the ENFRN learning to make an initial coarse selection of possible regulators for a specific gene, out of the whole set of genes present in a specific dataset. Later, the selected genes will be thoroughly checked using the remaining optimization phases of the ENFRN learning process to conclude the best regulators. The procedure follows three stages: in the first stage, each one of the *N *genes of the initial data is selected as output for the ENFRN network. All of the remaining *N*-1 genes are individually tested as possible regulators and we select the best *N*_*k *_candidate genes. The selection of the best possible regulators is implemented by means of a composite score (CS):(25)

where *n *is a constant ranging between 0 and 1, indicating the degree of importance we set for the network MSE value and regulation score *RS *(eq. 22). In this study the value of *n *is set to 0.6.

In the second stage, a set of all possible binary 2^*Nk *^configurations (where 1 represents the presence and 0 the absence of each one of the *N*_*k *_genes as input to the ENFRN) of the *N*_*k *_candidate genes is created. This choice is based on evidence from previous studies that genes might act individually as regulators of another gene or they might act concurrently with a group of genes to activate or repress a third-party gene or to achieve a specific biologic function [4]. Each one of these subsets of genes is inserted as input to the ENFRN and subsequently the best *N*_*B *_configurations are selected, in terms of network error. The two stages of the methodology described so far are based on the first phase of the ENFRN learning scheme (presented in section *Partitioning of Input and Output Space*), thus making use of the significant computational efficiency of this phase that allows a very large number of genes to be examined as possible regulators. In the last stage of the procedure, the *N*_*B *_selected subsets of candidate genes are optimized using the second and third phase of the ENFRN learning process and the final *N*_*F *_configurations are identified. These will be set as the potential regulators of the output gene. It must be underlined at this point that the procedure might yield a single potential regulator gene or the whole set of (maximum) *N*_*k *_genes, if indeed the expression patterns of these *N*_*k *_genes indicate them to be plausible regulators of the gene selected as output. The connectivity within the regulatory network is generally problem-dependent, however, based on biologically relevant evidence from previous studies we set the maximum number *N*_*k *_of possible regulators to 5 [4], unless otherwise stated.

## Authors' contributions

IAM conceived and implemented ENFRN and the methods for GRN reconstruction based on ENFRN. AD prepared the data sets and was responsible for writing the main body of the text. All of the above actions were supervised by DT. All authors read and approved the manuscript.

## Supplementary Material

Additional file 1 **In this file there are supplementary text, tables and figures describing full results of the proposed method on the three subsets of genes based on *cdc28 *dataset as well as results for the *alpha *datasets not presented in the main manuscript.** Additionally there are descriptions on the parameter values and intermediate results for the ENFRN models, as well as computational times for the methods used. Click here for file
