# Supplementary material for: Gene regulatory networks modelling using a dynamic evolutionary hybrid
Source: BMC Bioinformatics. 2010 Mar 18;11:140. doi: 10.1186/1471-2105-11-140 (PMC2848237; doi:10.1186/1471-2105-11-140)
Supplement: Additional file 1 — In this file there are supplementary text, tables and figures describing full results of the proposed method on the three subsets of genes based on cdc28 dataset as well as results for the alpha datasets not presented in the main manuscript. Additionally there are descriptions on the parameter values and intermediate results for the ENFRN models, as well as computational times for the methods used. [file 1471-2105-11-140-S1.PDF]

Gene Regulatory Networks Modelling using a Dynamic Evolutionary Hybrid

Ioannis A Maraziotis, Andrei Dragomir and Dimitris Thanos

Supplementary Material

In this paper we proposed a method for GRN reconstruction, based on a hybrid neural network structure named ENFRN. The inferred interactions originate from ENFRN structures. After those structures are trained on a specific dataset on a certain microarray experiment they can be tested against any other dataset (containing of course the same sets of genes) hence allowing to check for consistencies or inconsistencies among different biological experiments.

In this Supplementary Material we include several tables and figures concerning the parameters of the method, the inferred interactions, comparison with other methods in terms of GRN reconstruction and corresponding computational times. Given the stochastic nature of PSO, in all of the conducted experiments the simplification and training stages have been repeated for 5 times and the mean of the results are reported.

Tables (3,5,7,9,11) provide information regarding the extracted interactions. Those tables present the regulator and target genes, the composite score of the network on the test set used and the kind of biological knowledge (genetic, physical, intermediate, or none) that backs up the interaction. Tables (2,4,6,8,10) present the composite score levels at the 3 phases of learning and training, as well as the number of rules and output nodes in the initial and simplified structures. Figures (2,3,5) present the reconstructed networks when subset *cdc28* was used for testing in the three different data groups (8 histones, 19 genes group and Chen's – 41 genes, respectively).

As it can be noticed (see section *Determining Potential Regulators* section in the main manuscript), the composite score is an integrative measure indicating on the one hand the ability of the input variable(s) to predict the output signal and on the other the coherency concerning the expressional status alterations of output in comparison to the input (see *From ENFRN structure to Regulation Type* subsection in the main manuscript). The composite score of an interaction is the criterion based on which we decide whether or not we consider an interaction as valid. The lower the score of a certain interaction is, the more certain we can be of its validity.

In section 'Methods' of the main manuscript we describe the ENFRN training process composed of three learning phases. Those phases are initial structure creation, simplification phase and finally fine-tuning of the ENFRN weights and parameters. A general point of caution in every Neural Network based approach is the one concerning over-training of the network that can lead to over-fitting and thus to networks with poor generalization properties. Therefore, we have allowed a larger value of composite score during training, and thus ENFRN models manage to better fit the unseen data. Indeed as

we can depict in Fig. (4) in the majority of the cases the score of the extracted interaction is smaller in the testing set (mean composite score equal to 0.32) than the one of the training set (mean composite score equal to 0.30).

In Figure 6, we show the composite score distributions for the smallest (8 genes) and largest (41 genes) of the networks we reconstructed. As we can deduce from the figure the majority of the composite scores in the first case is between 0.5 and 0.6, while in the latter all the composite scores have values smaller than 0.5. However, as it can be noticed from the tables presenting the respective interactions, in both cases the vast majority of the inferred relations among genes correspond to biologically validated interactions. This indicates the flexibility of the proposed method over the input dataset. Indeed, it is shown that the method will adequately manage to determine the best interactions following the peculiarities of the input data, while the composite scores will allow the user to evaluate the significance of the inferred interactions and select the ones with the lower values.

A highly important aspect for every method applied to the problem at hand is the one concerning the time needed for reconstructing a certain network. We have mentioned that an advantage of the proposed methodology in terms of computational efficiency, concerns the first 2 stages of the general reconstructing framework described in section 'Determining Potential Regulators' and Figure 7 of the main manuscript. We have tested experimentally the computational efficiency of this part by randomly selecting sets of genes (from the same dataset we have used thought out this paper) whose number of members was ranging from 5 to 150 genes. We recorded the time it took the method to conclude the first 2 stages of the proposed reconstruction framework. Although, as we can deduce from Figure 7, the processing time follows an exponential increase with the size of genes sets, still the processing time is very low (it took less than 2.5 minutes to reconstruct the initial structure of 150 genes). These two stages of the reconstruction framework use the first phase of ENFRN training. Given that an ENFRN structure initially created by the first phase of the training process will be later pruned by the Structure Optimization phase we do not need to pay special attention in the parameters regarding the centers, widths and overlapping degrees of the fuzzy sets for the input and output variables. Hence we can set these parameters to low values (Table 1) to ensure an especially detailed initial structure (large number of rules and output nodes) that will be pruned later. The low values of these parameters will allow a coarse but accurate first estimate concerning the candidate regulators for every gene, which will be later refined.

Following we will discuss the total time the method needs to fully complete its operation and output the final reconstructed GRN, by commenting on the results presented in Table 12. Two especially important parameters of the proposed methodology are the number of BPSO and PSO particles used for simplification and fine-tuning. The larger the number of those particles the more time the algorithm will need to output the reconstructed network. In Table 12 we present results regarding the accuracy and computational processing time of the proposed methodology under different experimental setups regarding the two previously mentioned parameters, as well as comparisons to other methods. Prior continuing, we should note that timings are inevitably not only machine-dependent but also depended on various implementation and code optimizations techniques. Nevertheless, in Table 12 we present indicative of the relative timings, for the

algorithms we have used to compare our method with. All the methods (including ENFRN) have been implemented in Matlab© and run on a QUAD 2.6 GHz with 4 Gb RAM machine. As seen in Table 12, ENFNR based method had significantly better performance over the other algorithms in terms of both biological validity of the results and computational efficiency.

A first conclusion that can be drawn from Table 12 as well as Table 3 of the main manuscript, is that our method managed to significantly outperform all of the 3 methods we have used for comparison. Inspection of Table 12, proves that the proposed ENFRN-based approach is much more efficient than DN, DBN and RSONFIN in terms of computational time needed for the methods to fully reconstruct a certain GRN.

The results obtained when using 10 and 20 particles are similar both in outcome and time. This is mainly due to the number of iterations we allow PSO and BPSO to run without breaking their operation. By increasing this value and the number of particles we could end up with better values of training composite scores. A strategy such as this though would lead to overtraining of ENFRN and thus produce poor results when applied to unknown data.

The adequate results of the method, despite the small number of particles and training epochs for PSO (in comparison to other methods [4]) prove the efficiency of the algorithm responsible for creating the initial structure of ENFRN, as well as the proficient application of fuzzy logic and recurrent mechanism when applied to the intrinsic problem of GRN reconstruction.

As a final comment concerning the computational time needed for GRN reconstruction using the proposed approach, we should emphasize the fact that the method followed to infer the regulators of every gene present in a dataset can (in contrast to other methods like BNs and DBNs) be easily implemented using parallel techniques of processing. A framework such as this will allow the method to be feasibly applicable to much larger datasets.

**ENFRN Parameters**	**Values**
Minimum Distance for Rule Node Creation (F_{in})	0.01
Minimum Distance for Output Node Creation (F_{out})	0.01
Overlapping degree of input and output clusters (δ)	0.05
Number of particles for training in PSO	5
Number of particles for simplification in BPSO	5
Allowed Iterations for PSO and BPSO	20
Stop PSO and BPSO if no improvement after a number of iterations	5

Table 1: Values for the parameters of ENFRN models used in this study.

1. 8 Histones Group

A. *alpha*

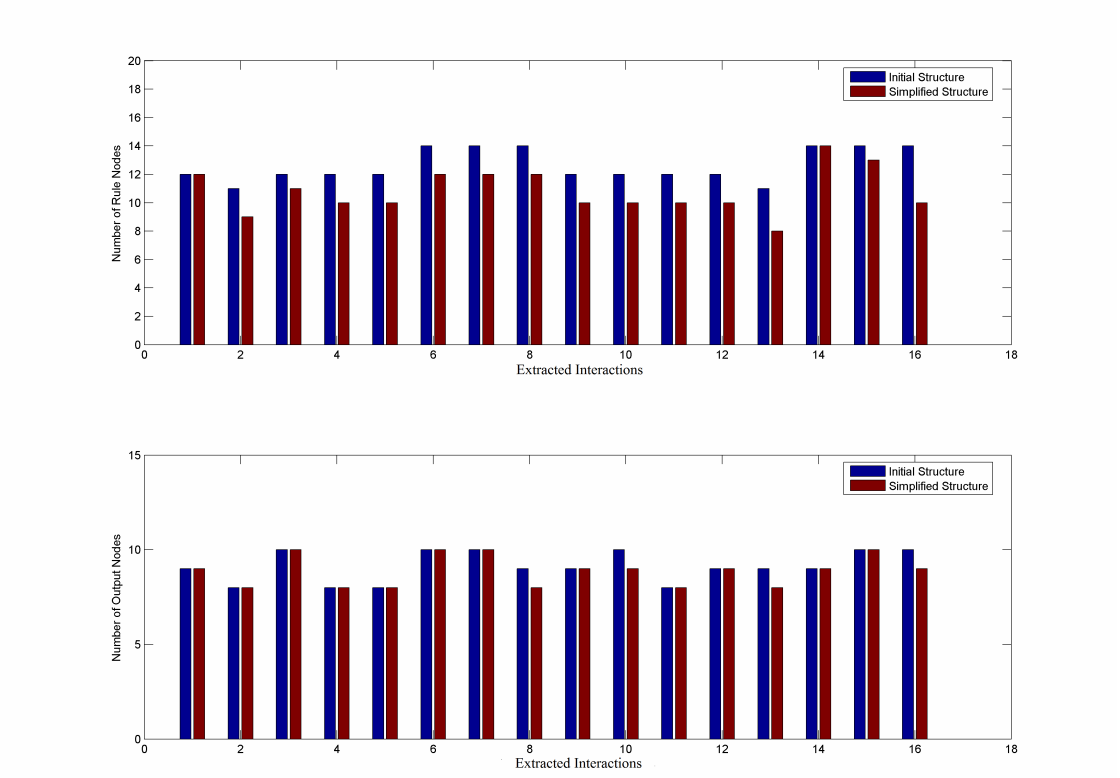

Figure 1: The number of rules and output nodes before and after the simplification process, based on the 16 ENFRN models describing the 16 interactions determined for the 8 histones, on *alpha* subset.

B. *cdc28*

a/a	Regulator	Target	Composite Error Values in ENFRN Structures			Number of Rules and Output Nodes in ENFRN Structures			
						Initial		Simplified	
			Initial	Simplified	Trained	Rules	Output	Rules	Output
1	HTB1	HTA1	0.899	0.935	0.533	12	9	12	9
2	HHF1	HTA1	1.127	1.127	0.712	10	9	10	9
3	HTB1	HHF1	0.842	0.815	0.536	12	8	10	8
4	HHF2	HTA1	1.127	1.127	0.712	10	9	10	9
5	HHF2	HTA2	0.657	0.669	0.543	14	10	12	10
6	HHT1	HTA1	0.898	0.916	0.664	12	9	10	9
7	HTB2	HHT1	0.877	0.877	0.743	11	10	10	10
8	HHT2	HTA1	1.127	1.127	0.712	10	9	10	9
9	HTB2	HHT2	0.981	0.981	0.673	11	9	10	9
10	HHF2	HHT2	0.890	0.881	0.589	14	10	13	10
11	HHT2	HHT1	0.962	0.975	0.749	14	10	10	9

Table 2: Information on ENFRN models that extracted the interactions

a/a	Regulator	Target	Type	Composite Score	Genetic Interaction	Physical Interaction	Intermediate Connection
1.	HTB1	HTA1	+	0.43403	1	1	-
2.	HHF1	HTA1	-	0.56429	1	1	-
3.	HTB1	HHF1	+	0.58183	1	1	-
4.	HHF2	HTA1	-	0.49662	1	1	-
5.	HHF2	HTA2	-	0.51812	0	1	-
6.	HHT1	HTA1	+	0.56794	1	1	-
7.	HTB2	HHT1	+	0.59228	0	1	-
8.	HHT2	HTA1	-	0.50463	1	1	-
9.	HTB2	HHT2	+	0.58642	0	0	-
10.	HHF2	HHT2	+	0.40183	1	1	-
11.	HHT2	HHT1	+	0.54196	1	1	-

Table 3: Interactions extracted

8 out of the 11 interactions have been identified as both genetic and physical interactions while 2 more as strictly physical, thus providing more than 90% of the interactions backed up by biological evidence.

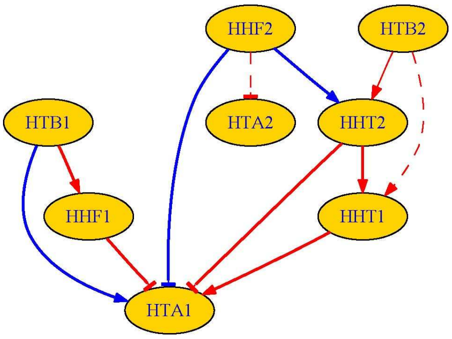

Figure 2: Extracted network for the 8 histones, based on *cdc28* test data set.

2. 19 genes Group

A. *Alpha*

a/a	Regulator	Target	Composite Score Values in ENFRN Structures			Number of Rules and Output Nodes in ENFRN Structures: Initial		Simplified	
			Initial	Simplified	Trained	Rules	Output	Rules	Output
1	CLB6	RAD53	1.015	1.015	0.428	2	2	2	2
2	CLB6	CLB5	1.33	1.33	0.491	3	2	3	2
3	POL12	MCD1	0.634	0.634	0.491	3	2	3	2
4	POL30	MSH2	0.799	0.769	0.741	11	6	7	6
5	PRI1	MSH2	0.518	0.393	0.819	19	13	15	10
6	MSH6	MSH2	0.729	0.447	0.587	18	12	15	11
7	POL12	CDC45	0.498	0.748	0.737	3	2	3	2
8	CDC45	CLB5	1.33	1.33	0.491	3	2	3	2
9	RAD53	PDS1	1.44	0.683	0.48	4	4	3	3
10	PDS1	CLB5	1.33	1.33	0.491	3	2	3	2
11	MCD1	PDS1	1.48	0.683	0.48	4	4	3	3
12	ASF1	MSH2	0.808	0.541	0.771	18	12	11	8
13	ASF1	CDC45	0.908	0.887	0.735	18	3	15	3
14	POL30	POL2	0.446	0.344	0.648	18	14	14	11
15	MSH6	POL2	0.446	0.344	0.648	18	14	14	11
16	PMS1	POL2	0.434	0.432	0.76	21	16	13	9
17	POL30	HPR5	1.18	1.03	0.82	11	4	6	4
18	PRI1	HPR5	0.79	0.995	0.782	15	5	11	4
19	RFA3	HPR5	0.56	0.75	0.65	16	8	14	8
20	PMS1	HPR5	0.599	0.582	0.65	15	7	12	7
21	PRI2	POL12	0.4923	0.4923	0.7185	13	7	13	7

Table 4: Information on ENFRN models that extracted the interactions

a/a	Regulator	Target	Type	Test Composite Score	Genetic Interaction	Physical Interaction	Intermediate Connection
1	CLB6	RAD53	+	0.36501	1	0	-
2	CLB6	CLB5	+	0.26469	1	0	-
3	POL12	MCD1	+	0.4641	0	0	-
4	POL30	MSH2	-	0.61536	1	1	-
5	PRI1	MSH2	-	0.525	1	0	-
6	MSH6	MSH2	+	0.43607	1	1	-
7	POL12	CDC45	+	0.56187	0	0	-
8	CDC45	CLB5	+	0.28331	1	0	-
9	RAD53	PDS1	+	0.35728	1	0	-
10	PDS1	CLB5	+	0.36518	1	1	-
11	MCD1	PDS1	+	0.37814	1	0	-
12	ASF1	MSH2	-	0.47243	1	0	-
13	ASF1	CDC45	-	0.65332	1	0	-
14	POL30	POL2	+	0.55725	1	0	-
15	MSH6	POL2	+	0.54639	1	0	-
16	PMS1	POL2	+	0.59536	1	0	-
17	POL30	HPR5	+	0.60493	1	1	-
18	PRI1	HPR5	+	0.64222	1	0	-
19	RFA3	HPR5	+	0.53237	0	0	-
20	PMS1	HPR5	+	0.61444	0	0	-
21	PRI2	POL12	+	0.59808	0	1	-

Table 5: Interactions extracted

B. *cdc28*

a/a	Regulator	Target	Composite Score Values in ENFRN Structures			Number of Rules and Output Nodes in ENFRN Structures			
						Initial		Simplified	
			Initial	Simplified	Trained	Rules	Output	Rules	Output
1	POL12	PRI2	0.952	0.952	0.710	3	2	3	2
2	CLB6	CLB5	1.330	1.330	0.491	3	2	3	2
3	POL12	MCD1	0.634	0.634	0.491	3	2	3	2
4	POL30	MSH2	0.799	0.769	0.741	11	6	7	6
5	PRI1	MSH2	0.518	0.393	0.819	19	13	15	10
6	MSH6	MSH2	0.729	0.447	0.587	18	12	15	11
7	CDC45	CLB5	1.330	1.330	0.491	3	2	3	2
8	PMS1	MSH2	0.830	0.717	0.577	15	11	13	10
9	RAD53	PDS1	2.840	0.683	0.480	4	4	3	3
10	PDS1	CLB5	1.330	1.330	0.491	3	2	3	2
11	MCD1	PDS1	2.160	0.585	0.455	5	5	4	4
12	ASF1	MSH2	0.729	0.447	0.587	18	12	15	11
13	PRI1	RAD54	0.612	0.661	0.983	20	12	14	8
14	CDC45	RAD54	0.976	0.974	0.898	15	11	14	11
15	ASF1	RAD54	0.976	0.974	0.898	15	11	14	11
16	POL30	POL2	0.446	0.344	0.648	18	14	14	11
17	MSH6	POL2	0.446	0.344	0.648	18	14	14	11
18	PMS1	POL2	0.434	0.432	0.760	21	16	13	9
19	RFA3	HPR5	0.560	0.750	0.650	16	8	14	8

Table 6: Information on ENFRN models that extracted the interactions

a/a	Regulator	Target	Type	Test Total Score	Genetic Interaction	Physical Interaction	Intermediate Connection
1.	POL12	PRI2	+	0.43466	0	1	-
2.	CLB6	CLB5	+	0.41068	1	0	-
3.	POL12	MCD1	+	0.39557	0	0	-
4.	POL30	MSH2	-	0.65296	1	1	-
5.	PRI1	MSH2	-	0.64373	1	0	-
6.	MSH6	MSH2	+	0.54989	1	1	-
7.	CDC45	CLB5	+	0.46271	1	0	-
8.	PMS1	MSH2	+	0.65298	1	1	-
9.	RAD53	PDS1	+	0.59583	1	0	-
10.	PDS1	CLB5	+	0.55971	1	1	-
11.	MCD1	PDS1	+	0.49503	1	0	-
12.	ASF1	MSH2	+	0.64932	1	0	-
13.	PRI1	RAD54	-	0.58412	1	0	-
14.	CDC45	RAD54	+	0.56406	1	0	-
15.	ASF1	RAD54	+	0.56405	1	0	-
16.	POL30	POL2	+	0.54795	1	0	-
17.	MSH6	POL2	+	0.61023	1	0	-
18.	PMS1	POL2	+	0.53604	1	0	-
19.	RFA3	HPR5	+	0.56684	0	0	2

Table 7: Interactions extracted

16 out of the 19 interactions have been identified as genetic interactions while 1 more as strictly physical, thus providing more than 90% of the interactions backed up by biological evidence.

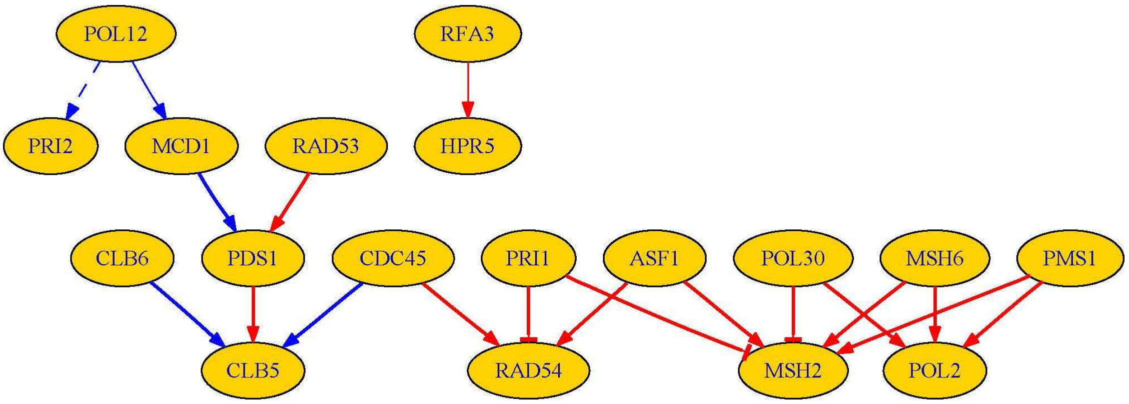

Figure 3: Extracted network for the 19 histones, based on *cdc28* test data set.

3. Chen's Data Set

A. *Alpha*

a/a	Regulator	Target	Composite Score Values in ENFRN Structures			Number of Rules and Output Nodes in ENFRN Structures Initial		Simplified	
			Initial	Simplified	Trained	Rules	Output	Rules	Output
1	SWI5	FKH1	0.351	0.383	0.306	20	13	15	10
2	SWI5	PCL2	0.277	0.283	0.283	14	13	6	6
3	STE12	ASH1	0.324	0.324	0.311	10	8	10	8
4	SPO12	DBF2	0.246	0.301	0.3	11	7	6	5
5	POG1	CLN1	0.384	0.346	0.342	19	13	11	9
6	HTB1	REM1	0.245	0.245	0.218	6	5	6	5
7	SWI4	CLN1	0.33	0.348	0.289	23	16	16	12
8	SWI4	SPO12	0.344	0.344	0.532	13	6	13	6
9	HHT1	HTA1	0.295	0.295	0.281	13	10	13	10
10	FKH1	HHT1	0.266	0.266	0.263	16	12	16	12
11	MOB1	CLB6	0.324	0.324	0.319	14	9	14	9
12	POG1	CLB2	0.327	0.337	0.337	10	8	8	7
13	CLN2	CLN3	0.323	0.373	0.37	10	9	9	8
14	CLN2	CLN1	0.33	0.348	0.289	23	16	16	12
15	MCM1	CLB6	0.32	0.32	0.365	14	10	14	10
16	SWI4	HTA1	0.238	0.284	0.284	13	9	10	8
17	MBP1	SKN7	0.283	0.283	0.317	12	7	12	7
18	NDD1	YHP1	0.277	0.277	0.341	15	8	15	8
19	OPY2	CDC20	0.334	0.334	0.406	14	9	14	9
20	MBP1	SWI4	0.313	0.313	0.339	12	9	12	9
21	FKH1	HTA1	0.262	0.262	0.279	16	11	16	11
22	CLN3	YHP1	0.284	0.284	0.309	14	8	14	8
23	CLN1	SIC1	0.304	0.273	0.303	21	12	16	12
24	PCL9	SKN7	0.253	0.233	0.232	14	8	9	7
25	MCM1	YOX1	0.363	0.377	0.372	14	10	11	8
26	POG1	CLN2	0.214	0.214	0.268	19	9	19	9
27	POG1	ASH1	0.253	0.253	0.237	19	10	19	10
28	HCM1	MCM1	0.283	0.283	0.54	17	8	17	8
29	SWI5	CLB2	0.454	0.454	0.161	2	2	2	2
30	FKH2	CDC20	0.395	0.395	0.337	14	10	14	10
31	PCL7	CLB2	0.385	0.327	0.327	13	10	4	4
32	YHP1	SWE1	0.262	0.262	0.304	13	10	13	10
33	SWE1	MFA2	0.283	0.359	0.359	8	8	7	7
34	CLB2	FKH1	0.351	0.383	0.306	20	13	15	10
35	MCM1	YHP1	0.437	0.362	0.362	7	6	6	5
36	SWI4	SWI5	0.311	0.311	0.308	13	11	13	11
37	FAR1	CLB1	0.398	0.398	0.442	14	12	14	12
38	SIC1	SVS1	0.247	0.247	0.331	13	10	13	10

39	PCL2	SWI5	0.325	0.351	0.351	13	11	10	9
40	CLN2	SWI4	0.27	0.266	0.29	22	14	17	14
41	DBF2	SWI5	0.282	0.276	0.322	23	17	16	13
42	CLB2	SWE1	0.387	0.449	0.449	10	6	6	5
43	CLB2	NDD1	0.321	0.284	0.284	16	10	11	8
44	ASH1	HCM1	0.288	0.288	0.4	11	10	11	10
45	CLB1	CLN2	0.012	0.012	0.01	2	2	2	2
46	PCL9	HCM1	0.308	0.308	0.414	14	9	14	9
47	SWI4	PCL7	0.283	0.299	0.299	14	12	5	5
48	CLB1	ASH1	0.097	0.097	0.086	2	2	2	2
49	FAR1	CLB2	0.476	0.539	0.539	5	5	4	4
50	NDD1	FKH2	0.431	0.431	0.329	15	9	15	9
51	SIM1	HHT1	0.253	0.253	0.285	17	11	17	11
52	HTA1	SKN7	0.252	0.252	0.275	12	9	12	9
53	SIC1	CLN3	0.265	0.278	0.293	22	14	18	11
54	CDC20	CLB2	0.345	0.301	0.301	12	10	9	7
55	CLN1	SWE1	0.322	0.322	0.247	16	11	16	11
56	FKH1	FKH2	0.366	0.395	0.39	16	10	11	8
57	SVS1	SKN7	0.245	0.245	0.295	15	10	15	10
58	CLB2	HCM1	0.338	0.373	0.373	10	7	5	5
59	CLN3	CLN1	0.33	0.348	0.289	23	16	16	12
60	PCL2	CLB4	0.277	0.364	0.364	12	8	6	4
61	FKH1	SWE1	0.263	0.263	0.303	16	13	16	13
62	CLB1	HCM1	0.106	0.106	0.091	2	2	2	2
63	SPO12	CLN2	0.211	0.211	0.313	11	8	11	8
64	FKH2	SPO12	0.335	0.335	0.407	14	7	14	7
65	SWI5	SIC1	0.314	0.332	0.401	22	12	16	9
66	CLB2	FKH2	0.3	0.31	0.352	21	12	12	8
67	FKH2	SWI5	0.282	0.276	0.322	23	17	16	13
68	CLB1	CLB2	0.437	0.437	0.137	2	2	2	2
69	HHT1	SWE1	0.248	0.248	0.274	13	9	13	9
70	CLB2	POG1	0.302	0.323	0.323	19	13	12	8
71	DBF2	CDC20	0.419	0.586	0.586	9	5	4	3
72	MFA1	CLB4	0.273	0.273	0.542	8	6	8	6
73	HHT1	MCM1	0.25	0.25	0.293	13	7	13	7
74	HCM1	PCL2	0.345	0.345	0.38	17	9	17	9
75	ASH1	CLB2	0.335	0.368	0.368	12	9	5	5
76	REM1	NDD1	0.245	0.245	0.327	11	9	11	9
77	CDC20	SIC1	0.304	0.273	0.303	21	12	16	12
78	DBF2	MOB1	0.293	0.403	0.402	10	8	5	4
79	CLB2	REM1	0.276	0.324	0.322	12	8	8	6
80	FKH2	ASH1	0.271	0.271	0.314	14	11	14	11
81	CLB1	SVS1	0.069	0.069	0.069	2	2	2	2
82	SWE1	SIC1	0.38	0.441	0.439	15	11	8	7
83	CLB6	CLN3	0.301	0.301	0.303	16	10	16	10

Table 8: Information on ENFRN models that extracted the interactions

a/a	Regulator	Target	Type	Test Composite Score	Genetic Interaction	Physical Interaction	Intermediate Connection
1	SWI5	FKH1	+	0.28839	1	0	-
2	SWI5	PCL2	+	0.37271	0	1	-
3	STE12	ASH1	+	0.27529	1	0	-
4	SPO12	DBF2	+	0.31364	1	0	-
5	POG1	CLN1	-	0.37537	1	0	-
6	HTB1	REM1	+	0.23275	0	0	-
7	SWI4	CLN1	+	0.26917	1	0	-
8	SWI4	SPO12	-	0.35007	0	0	1
9	HHT1	HTA1	+	0.28732	1	1	-
10	FKH1	HHT1	+	0.27241	0	0	-
11	MOB1	CLB6	+	0.27041	0	0	-
12	POG1	CLB2	+	0.32732	0	1	-
13	CLN2	CLN3	-	0.37156	1	0	-
14	CLN2	CLN1	+	0.26476	1	0	-
15	MCM1	CLB6	+	0.26709	0	0	-
16	SWI4	HTA1	+	0.25682	1	0	-
17	MBP1	SKN7	+	0.29724	1	1	-
18	NDD1	YHP1	+	0.28438	0	0	-
19	OPY2	CDC20	+	0.28929	0	0	-
20	MBP1	SWI4	-	0.32066	1	0	-
21	FKH1	HTA1	+	0.24046	0	0	-
22	CLN3	YHP1	+	0.28753	0	0	-
23	CLN1	SIC1	+	0.30543	1	1	-
24	PCL9	SKN7	+	0.25134	0	0	-
25	MCM1	YOX1	-	0.34596	0	1	-
26	POG1	CLN2	+	0.23723	0	0	-
27	POG1	ASH1	+	0.25454	0	0	-
28	HCM1	MCM1	+	0.25217	0	0	-
29	SWI5	CLB2	+	0.17348	1	1	-
30	FKH2	CDC20	-	0.38557	1	0	-
31	PCL7	CLB2	+	0.38506	0	1	-
32	YHP1	SWE1	+	0.25162	0	0	-
33	SWE1	MFA2	+	0.28116	0	1	-
34	CLB2	FKH1	+	0.27734	1	0	-
35	MCM1	YHP1	+	0.43679	0	1	-
36	SWI4	SWI5	-	0.32049	1	0	-
37	FAR1	CLB1	+	0.44146	0	0	2
38	SIC1	SVS1	+	0.2578	0	0	-
39	PCL2	SWI5	+	0.32467	0	1	-
40	CLN2	SWI4	+	0.26381	1	0	-
41	DBF2	SWI5	-	0.30345	1	0	-
42	CLB2	SWE1	+	0.38686	0	1	-
43	CLB2	NDD1	+	0.43184	0	1	-
44	ASH1	HCM1	+	0.27064	0	0	-
45	CLB1	CLN2	+	0.02778	0	0	-
46	PCL9	HCM1	+	0.31231	0	0	2
47	SWI4	PCL7	+	0.27128	0	1	-
48	CLB1	ASH1	+	0.02783	0	0	-

49	FAR1	CLB2	+	0.47576	0	1	-
50	NDD1	FKH2	-	0.39547	1	1	-
51	SIM1	HHT1	+	0.26507	0	0	-
52	HTA1	SKN7	+	0.28093	0	0	-
53	SIC1	CLN3	-	0.29585	1	1	-
54	CDC20	CLB2	+	0.34488	0	1	-
55	CLN1	SWE1	+	0.29513	1	0	-
56	FKH1	FKH2	-	0.38863	1	0	-
57	SVS1	SKN7	+	0.26282	0	0	-
58	CLB2	HCM1	+	0.33834	0	1	-
59	CLN3	CLN1	+	0.27146	1	0	-
60	PCL2	CLB4	+	0.35189	0	0	2
61	FKH1	SWE1	+	0.25435	0	0	-
62	CLB1	HCM1	+	0.02046	0	0	2
63	SPO12	CLN2	+	0.23563	0	0	1
64	FKH2	SPO12	+	0.34023	0	0	1
65	SWI5	SIC1	+	0.35223	1	0	-
66	CLB2	FKH2	+	0.30509	1	1	-
67	FKH2	SWI5	-	0.30795	1	0	-
68	CLB1	CLB2	+	0.07883	1	1	-
69	HHT1	SWE1	+	0.22974	0	0	-
70	CLB2	POG1	+	0.37187	0	1	-
71	DBF2	CDC20	+	0.41944	0	1	-
72	MFA1	CLB4	+	0.28162	0	0	-
73	HHT1	MCM1	+	0.2581	0	0	-
74	HCM1	PCL2	+	0.37284	0	0	2
75	ASH1	CLB2	+	0.33469	0	1	-
76	REM1	NDD1	+	0.255	0	0	-
77	CDC20	SIC1	+	0.30354	1	0	-
78	DBF2	MOB1	-	0.42495	1	1	-
79	CLB2	REM1	-	0.34541	1	0	-
80	FKH2	ASH1	+	0.28332	0	0	-
81	CLB1	SVS1	+	0.03096	0	0	-
82	SWE1	SIC1	-	0.38071	1	0	-
83	CLB6	CLN3	-	0.30596	1	0	-

Table 9: Interactions extracted

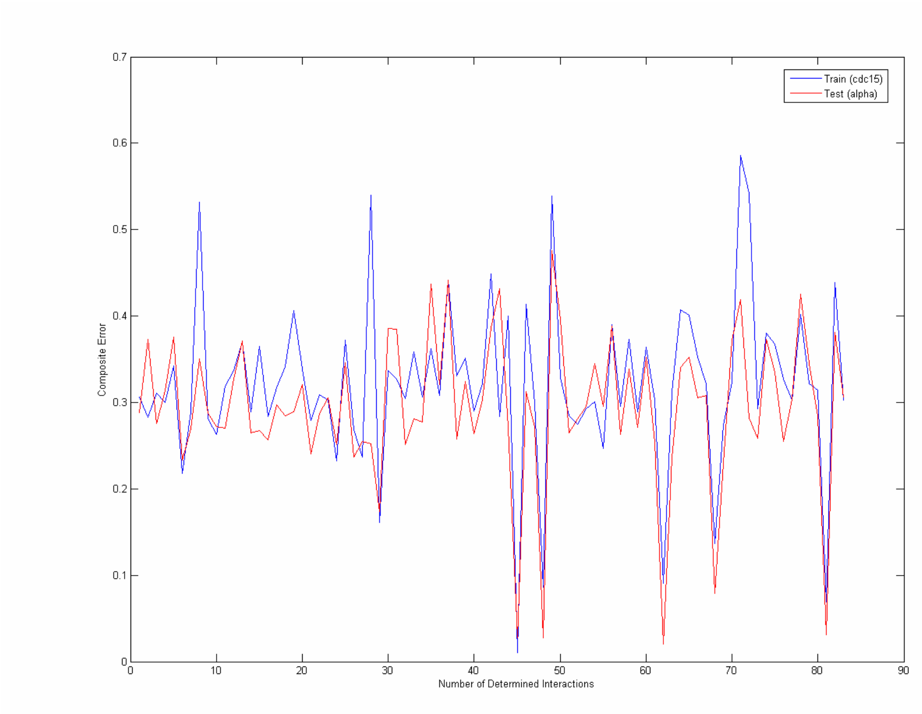

Figure 4: Test Vs Training composite scores for Chen's Data set – testing in *alpha* data set.

B. *cdc28*

a/a	Reg	Targ	Composite Score Values in ENFRN Structures			Number of Rules and Output Nodes in ENFRN Structures			
						Initial		Simplified	
			Initial	Simplified	Trained	Rules	Output	Rules	Output
1	DBF2	CDC20	0.419	0.586	0.586	9	5	4	3
2	PCL2	SWI5	0.325	0.351	0.351	13	11	10	9
3	SWI4	CLN1	0.33	0.348	0.289	23	16	16	12
4	HHT1	HTA1	0.295	0.295	0.281	13	10	13	10
5	FKH2	CDC20	0.395	0.395	0.337	14	10	14	10
6	MBP1	SWI4	0.313	0.313	0.339	12	9	12	9
7	CDC20	CLB2	0.345	0.301	0.301	12	10	9	7
8	CLN2	SWI4	0.27	0.266	0.29	22	14	17	14
9	SWI4	HTA1	0.238	0.284	0.284	13	9	10	8
10	ASH1	HCM1	0.288	0.288	0.4	11	10	11	10
11	POG1	CLN1	0.384	0.346	0.342	19	13	11	9
12	SIM1	MOB1	0.278	0.278	0.273	17	11	17	11
13	POG1	ASH1	0.253	0.253	0.237	19	10	19	10
14	CLB1	CLB2	0.437	0.437	0.137	2	2	2	2
15	SWI5	FKH1	0.318	0.318	0.32	17	12	17	12
16	PCL9	HCM1	0.308	0.308	0.414	14	9	14	9
17	DBF2	MOB1	0.293	0.403	0.402	10	8	5	4

18	SWI5	SIC1	0.314	0.332	0.401	22	12	16	9
19	SPO12	DBF2	0.246	0.301	0.3	11	7	6	5
20	SIC1	SVS1	0.247	0.247	0.331	13	10	13	10
21	MBP1	SKN7	0.283	0.283	0.317	12	7	12	7
22	MCM1	YOX1	0.363	0.377	0.372	14	10	11	8
23	CLB2	SWE1	0.387	0.449	0.449	10	6	6	5
24	SPO12	CLN2	0.211	0.211	0.313	11	8	11	8
25	SWI5	PCL2	0.277	0.283	0.283	14	13	6	6
26	SWI4	PCL7	0.283	0.299	0.299	14	12	5	5
27	MFA1	MBP1	0.318	0.318	0.548	8	5	8	5
28	CLB1	CLN2	0.012	0.012	0.01	2	2	2	2
29	CLB1	SVS1	0.069	0.069	0.069	2	2	2	2
30	SWI4	FAR1	0.309	0.309	0.355	13	11	13	11
31	FKH1	HTA1	0.262	0.262	0.279	16	11	16	11
32	FKH1	HHT1	0.266	0.266	0.263	16	12	16	12
33	CLB2	NDD1	0.321	0.284	0.284	16	10	11	8
34	SWE1	MFA2	0.283	0.359	0.359	8	8	7	7
35	POG1	MNN1	0.258	0.258	0.29	19	14	19	14
36	HCM1	PCL2	0.345	0.345	0.38	17	9	17	9
37	CLB2	HCM1	0.338	0.373	0.373	10	7	5	5
38	CLB2	FKH1	0.351	0.383	0.306	20	13	15	10
39	STE12	ASH1	0.324	0.324	0.311	10	8	10	8
40	POG1	CLB2	0.327	0.337	0.337	10	8	8	7
41	CLB2	POG1	0.302	0.323	0.323	19	13	12	8
42	FKH1	FKH2	0.366	0.395	0.39	16	10	11	8
43	CLN3	CLN1	0.33	0.348	0.289	23	16	16	12
44	CLN2	CLN1	0.33	0.348	0.289	23	16	16	12
45	CLB6	CLN3	0.301	0.301	0.303	16	10	16	10
46	PCL9	SKN7	0.253	0.233	0.232	14	8	9	7
47	CLB1	ASH1	0.097	0.097	0.086	2	2	2	2
48	CLB2	FKH2	0.3	0.31	0.352	21	12	12	8
49	ASH1	CLB2	0.335	0.368	0.368	12	9	5	5
50	CLN2	CLN3	0.323	0.373	0.37	10	9	9	8
51	CLN3	YHP1	0.284	0.284	0.309	14	8	14	8
52	SWE1	SIC1	0.38	0.441	0.439	15	11	8	7
53	NDD1	FKH2	0.431	0.431	0.329	15	9	15	9
54	CLB2	REM1	0.276	0.324	0.322	12	8	8	6
55	CLN2	FAR1	0.439	0.439	0.634	10	8	10	8
56	PCL7	CLB2	0.385	0.327	0.327	13	10	4	4
57	HTB1	REM1	0.245	0.245	0.218	6	5	6	5
58	CDC20	SIC1	0.304	0.273	0.303	21	12	16	12
59	FAR1	CLB2	0.476	0.539	0.539	5	5	4	4
60	MCM1	YHP1	0.437	0.362	0.362	7	6	6	5
61	CLB1	HCM1	0.106	0.106	0.091	2	2	2	2
62	SWI4	SWI5	0.311	0.311	0.308	13	11	13	11
63	FKH2	SWI5	0.282	0.276	0.322	23	17	16	13
64	PCL2	CLB4	0.277	0.364	0.364	12	8	6	4
65	CLN1	SWE1	0.322	0.322	0.247	16	11	16	11
66	FKH2	ASH1	0.271	0.271	0.314	14	11	14	11
67	CLN1	SIC1	0.304	0.273	0.303	21	12	16	12
68	DBF2	SWI5	0.282	0.276	0.322	23	17	16	13
69	SIC1	CLN3	0.265	0.278	0.293	22	14	18	11
70	SWI5	CLB2	0.454	0.454	0.161	2	2	2	2

Table 10: Information on ENFRN models that extracted the interactions

a/a	Regulator	Target	Type	Test Composite Score	Genetic Interaction	Physical Interaction	Intermediate Connection
1	DBF2	CDC20	+	0.41944	0	1	-
2	PCL2	SWI5	+	0.32467	0	1	-
3	SWI4	CLN1	+	0.27004	1	0	-
4	HHT1	HTA1	+	0.35928	1	1	-
5	FKH2	CDC20	-	0.49529	1	0	-
6	MBP1	SWI4	-	0.32922	1	0	-
7	CDC20	CLB2	+	0.34488	0	1	-
8	CLN2	SWI4	+	0.28629	1	0	-
9	SWI4	HTA1	+	0.32997	1	0	-
10	ASH1	HCM1	+	0.27872	0	0	-
11	POG1	CLN1	-	0.39697	1	0	-
12	SIM1	MOB1	+	0.28704	0	0	-
13	POG1	ASH1	+	0.2528	0	0	-
14	CLB1	CLB2	+	0.14843	1	1	-
15	SWI5	FKH1	-	0.4683	1	0	-
16	PCL9	HCM1	+	0.35165	0	0	2
17	DBF2	MOB1	-	0.47922	1	1	-
18	SWI5	SIC1	+	0.45409	1	0	-
19	SPO12	DBF2	+	0.39346	1	0	-
20	SIC1	SVS1	+	0.28185	0	0	-
21	MBP1	SKN7	+	0.31302	1	1	-
22	MCM1	YOX1	-	0.45419	0	1	-
23	CLB2	SWE1	+	0.38686	0	1	-
24	SPO12	CLN2	+	0.3465	0	0	1
25	SWI5	PCL2	+	0.37271	0	1	-
26	SWI4	PCL7	+	0.27128	0	1	-
27	MFA1	MBP1	+	0.2722	0	0	-
28	CLB1	CLN2	+	0.14985	0	0	-
29	CLB1	SVS1	+	0.07284	0	0	-
30	SWI4	FAR1	+	0.30995	0	0	1
31	FKH1	HTA1	+	0.28757	0	0	-
32	FKH1	HHT1	+	0.28763	0	0	-
33	CLB2	NDD1	+	0.43184	0	1	-
34	SWE1	MFA2	+	0.28116	0	1	-
35	POG1	MNN1	+	0.25307	0	0	-
36	HCM1	PCL2	+	0.41778	0	0	2
37	CLB2	HCM1	+	0.33834	0	1	-
38	CLB2	FKH1	+	0.38064	1	0	-
39	STE12	ASH1	+	0.28034	1	0	-
40	POG1	CLB2	+	0.32732	0	1	-
41	CLB2	POG1	+	0.37187	0	1	-
42	FKH1	FKH2	-	0.4278	1	0	-
43	CLN3	CLN1	+	0.27198	1	0	-
44	CLN2	CLN1	+	0.27258	1	0	-
45	CLB6	CLN3	-	0.3333	1	0	-
46	PCL9	SKN7	+	0.26794	0	0	-
47	CLB1	ASH1	+	0.02996	0	0	-
48	CLB2	FKH2	+	0.36824	1	1	-
49	ASH1	CLB2	+	0.33469	0	1	-

50	CLN2	CLN3	-	0.39634	1	0	-
51	CLN3	YHP1	+	0.28212	0	0	-
52	SWE1	SIC1	-	0.45977	1	0	-
53	NDD1	FKH2	-	0.48611	1	1	-
54	CLB2	REM1	-	0.34958	1	0	-
55	CLN2	FAR1	+	0.46377	1	1	-
56	PCL7	CLB2	+	0.38506	0	1	-
57	HTB1	REM1	+	0.26291	0	0	-
58	CDC20	SIC1	+	0.40372	1	0	-
59	FAR1	CLB2	+	0.47576	0	1	-
60	MCM1	YHP1	+	0.43679	0	1	-
61	CLB1	HCM1	+	0.05617	0	0	2
62	SWI4	SWI5	-	0.34698	1	0	-
63	FKH2	SWI5	-	0.3482	1	0	-
64	PCL2	CLB4	+	0.41949	0	0	2
65	CLN1	SWE1	+	0.40375	1	0	-
66	FKH2	ASH1	+	0.28992	0	0	-
67	CLN1	SIC1	+	0.40008	1	1	-
68	DBF2	SWI5	-	0.34448	1	0	-
69	SIC1	CLN3	-	0.32159	1	1	-
70	SWI5	CLB2	+	0.19419	1	1	-

Table 11: Interactions extracted

We can deduce from Table 11 that the method has extracted 33 genetic interactions, and 16 solely physical interactions, 6 intermediate. So we have either 70% or 79% (with intermediate) from the interactions extracted to be in accordance with prior biological knowledge.

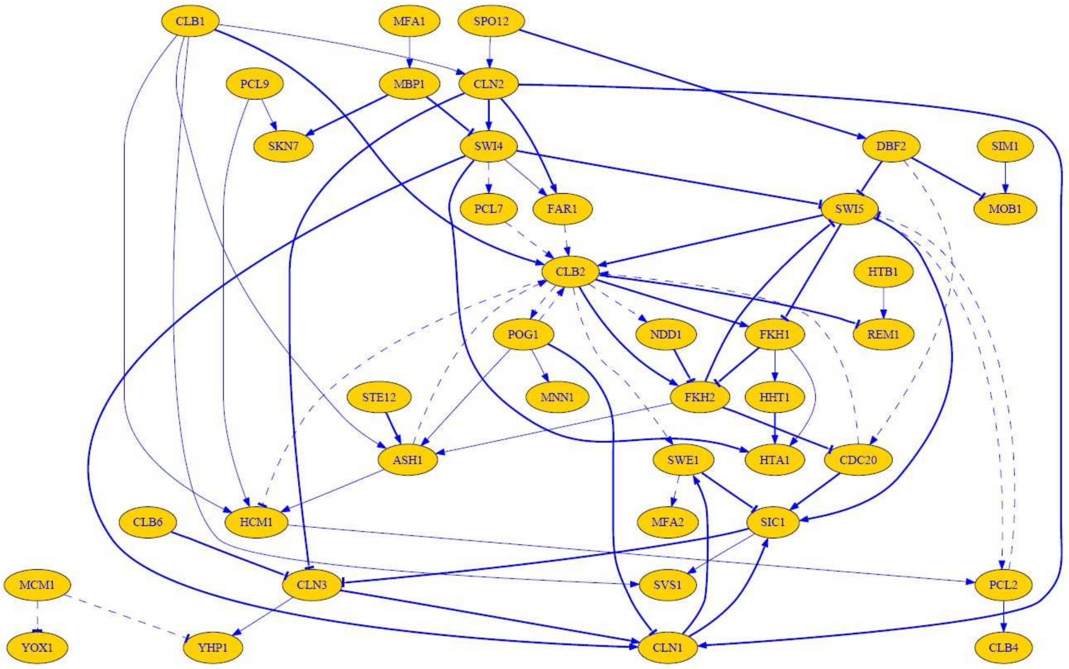

Figure 5: Extracted network for the Chen's data, based on *cdc28* test data set.

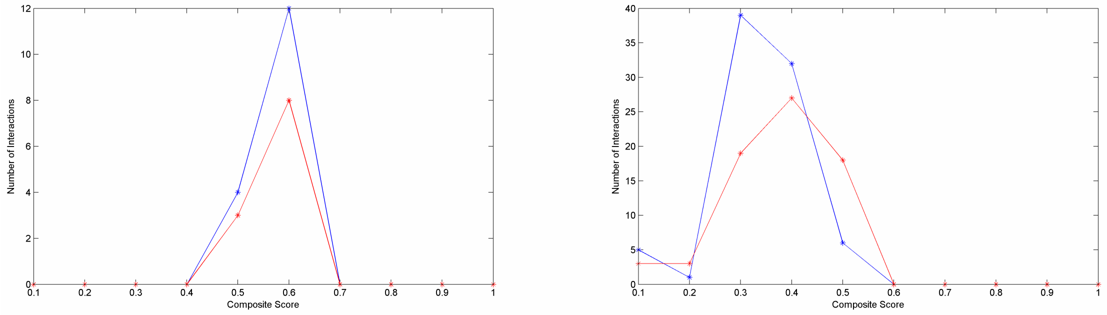

Figure 6: Distribution of Composite Scores corresponding to the smallest (8 genes - left) and largest (41 genes - right) of all datasets we have used to reconstruct the corresponding GRNs. In both cases presented in this figure the red lines correspond to the test composite scores of the ENFRN structures using the *alpha* dataset while the blue when using the *cdc28* subset.

Modeling Approach	Particles used in BPSO (structure simplification)	Particles used in PSO (parameters fine-tuning)	Avg of Composite Score	True Positives	True Positives (Intermediate gene)	False Positives	Time (mins)
ENFRN	5	5	0.456	15	3	3	6
	10	10	0.412	12	5	2	9
	15	15	0.390	12	4	2	12
RSONFIN	-	-	-	5	5	6	22
Bayesian Network	-	-	-	3	4	7	29
Dynamic Bayesian Network	-	-	-	5	4	4	31

Table 12: Comparison in terms of accuracy and computational time of the proposed method against 3 other methods. We also check against different parameter setups for ENFRN. For the results of this table we have used *alpha* subset as testing dataset.

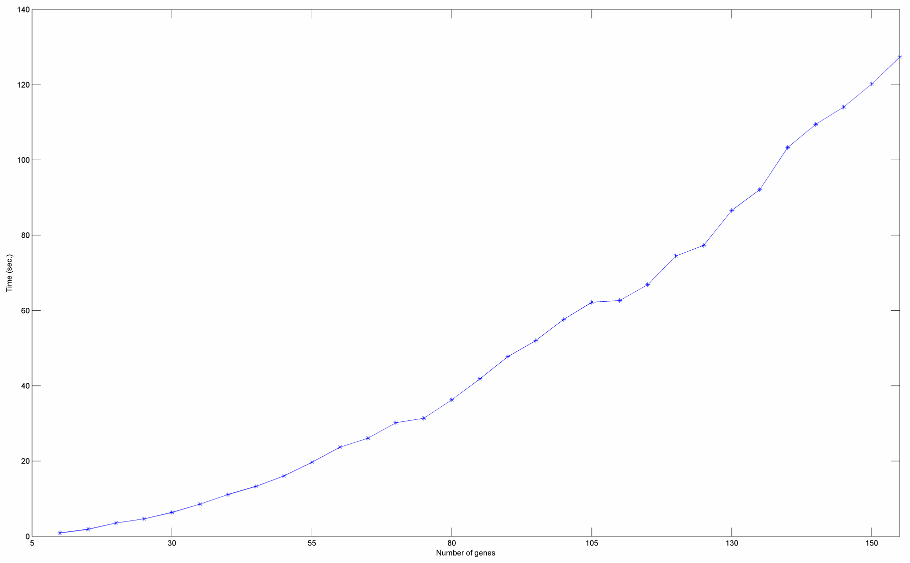

Figure 7: Time needed for the 1st phase of the proposed ENFRN-based methodology to roughly select the best sets of regulators for all genes in a specific dataset. The x-axis represents the subsets of genes selected for reconstructing the corresponding networks ranging from 5 to 150 genes with a step of increase of 5.As we can see, despite the fact that the method follows an exponential growth in the time needed, it took less than 2.5 minutes to finish the coarse reconstruction of a subset containing 150 genes.
